# MBNL splicing factors regulate the microtranscriptome of skeletal muscles

**DOI:** 10.1093/nar/gkae774

**Published:** 2024-09-11

**Authors:** Agnieszka Piasecka, Michał W Szcześniak, Michał Sekrecki, Arkadiusz Kajdasz, Łukasz J Sznajder, Anna Baud, Krzysztof Sobczak

**Affiliations:** Laboratory of Gene Therapy, Department of Gene Expression, Institute of Molecular Biology and Biotechnology, Faculty of Biology, Adam Mickiewicz University, Uniwersytetu Poznanskiego 6, 61-614 Poznań, Poland; Institute of Human Biology and Evolution, Faculty of Biology, Adam Mickiewicz University, Uniwersytetu Poznanskiego 6, 61-614 Poznań, Poland; Laboratory of Gene Therapy, Department of Gene Expression, Institute of Molecular Biology and Biotechnology, Faculty of Biology, Adam Mickiewicz University, Uniwersytetu Poznanskiego 6, 61-614 Poznań, Poland; Laboratory of Gene Therapy, Department of Gene Expression, Institute of Molecular Biology and Biotechnology, Faculty of Biology, Adam Mickiewicz University, Uniwersytetu Poznanskiego 6, 61-614 Poznań, Poland; Laboratory of Bioinformatics, Institute of Bioorganic Chemistry, Polish Academy of Sciences, Noskowskiego 12/14, 61-704Poznań, Poland; Laboratory of Gene Therapy, Department of Gene Expression, Institute of Molecular Biology and Biotechnology, Faculty of Biology, Adam Mickiewicz University, Uniwersytetu Poznanskiego 6, 61-614 Poznań, Poland; Department of Chemistry and Biochemistry, University of Nevada, Las Vegas, NV 89154, USA; Laboratory of Gene Therapy, Department of Gene Expression, Institute of Molecular Biology and Biotechnology, Faculty of Biology, Adam Mickiewicz University, Uniwersytetu Poznanskiego 6, 61-614 Poznań, Poland; Laboratory of Gene Therapy, Department of Gene Expression, Institute of Molecular Biology and Biotechnology, Faculty of Biology, Adam Mickiewicz University, Uniwersytetu Poznanskiego 6, 61-614 Poznań, Poland

## Abstract

Muscleblind like splicing regulators (MBNLs) govern various RNA-processing steps, including alternative splicing, polyadenylation, RNA stability and mRNA intracellular localization. In myotonic dystrophy type 1 (DM1), the most common muscular dystrophy in adults, MBNLs are sequestered on toxic RNA containing expanded CUG repeats, which leads to disruption of MBNL-regulated processes and disease features of DM1. Herein, we show the significance of MBNLs in regulating microtranscriptome dynamics during the postnatal development of skeletal muscles and in microRNA (miRNA) misregulation observed in mouse models and patients with DM1. We identify multiple miRNAs sensitive to MBNL proteins insufficiency and reveal that many of them were postnatally regulated, which correlates with increases in the activity of these proteins during this process. In adult Mbnl1-knockout mice, miRNA expression exhibited an adult-to-newborn shift. We hypothesize that Mbnl1 deficiency influences miRNA levels through a combination of mechanisms. First, the absence of Mbnl1 protein results in alterations to the levels of pri-miRNAs. Second, MBNLs affect miRNA biogenesis by regulating the alternative splicing of miRNA primary transcripts. We propose that the expression of miR-23b, miR-27b and miR-24-1, produced from the same cluster, depends on the MBNL-sensitive inclusion of alternative exons containing miRNA sequences. Our findings suggest that MBNL sequestration in DM1 is partially responsible for altered miRNA activity. This study provides new insights into the biological roles and functions of MBNL proteins as regulators of miRNA expression in skeletal muscles.

## Graphical Abstract

**Graphical Abstract:**
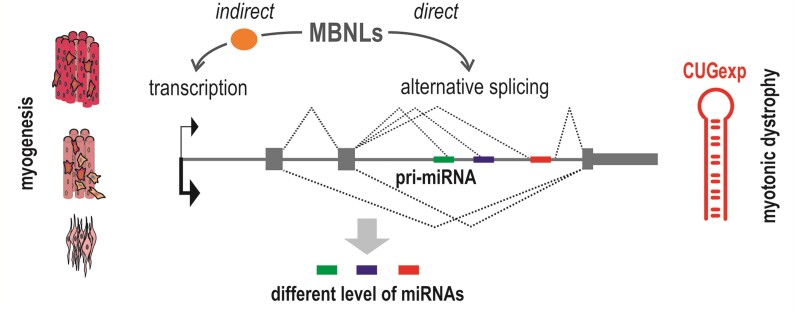

## Introduction

Muscleblind like splicing regulators (MBNLs) belong to the family of tissue-specific RNA binding proteins (RBPs), which influence alternative splicing (AS), alternative polyadenylation (APA), mRNA stability and trafficking (1–7). In mammals, MBNLs are encoded by three paralogs, *MBNL1*, *MBNL2* and *MBNL3*, and share sequence and structural similarities, including the presence of four zinc fingers (ZnFs), domains crucial for recognition of consensus sequences in pre-mRNA and mRNA targets that are common to all MBNLs. They differ in cellular localization, multimerization capacity, affinity to RNA sequence motifs and AS activity (8,9). The activity of MBNL paralogs depends on their expression level in specific tissues and developmental stages (2,3). MBNL1 is involved in embryonic stem cell differentiation as well as muscular and immune system development (10–12). MBNL2 is mostly expressed in the brain (13), whereas the MBNL3 expression level is low in the majority of adult tissues (14) but high in the placenta (15).

Myotonic dystrophy type 1 (DM1) is the most common form of adult muscular dystrophy and results from a CTG repeat tract expansion in the 3′ untranslated region (3′UTR) of the dystrophia myotonica protein kinase (*DMPK*) gene (16). The disease is characterized by multiorgan dysfunction with prevalent manifestations within skeletal muscles, the heart, and the neuronal system. The toxic agent in DM1 is a DMPK mRNA that contains expanded CUG repeats (CUG^exp^), interacts with many proteins and is retained in the nucleus, forming RNA foci (17). The CUG^exp^ RNA sequesters MBNL proteins, leading to the disruption of many processes regulated by these proteins and further to disease-associated phenotypes, such as myotonia (caused by CLCN1 missplicing, (18) muscle weakness and insulin resistance (19) or cardiac abnormalities, including fibrosis and conduction anomalies (20,21). Abnormalities in AS and APA are the processes best described in DM (22,23); however, some studies have also shown quantitative changes in microRNAs (miRNAs) (24–27).

Similar to that of protein-coding genes, the expression of miRNAs is regulated at both the transcriptional and posttranscriptional levels by multiple RBPs (28). MiRNA genes are first transcribed in a manner similar to that of pre-mRNAs and then go through a series of sequential steps involving two major endoribonucleases, Drosha and Dicer. The processing of the primary transcript of miRNA (pri-miRNA) by Drosha in the nucleus leads to the generation of an ∼60–70 nucleotide-long precursor, pre-miRNA. Then, in the cytoplasm, Dicer produces ∼22 nucleotide-long mature miRNAs. The rate of transcription seems to play a key role in establishing mature miRNA levels (29–31). However, recent studies have also revealed multiple posttranscriptional mechanisms that may regulate miRNA processing in a gene-specific or global manner. Sequence-specific RBPs can positively or negatively regulate miRNA processing through binding to a pri-miRNA or to a pre-miRNA (32–40). Moreover, global miRNA levels can be altered by modulating the activity of miRNA core processing factors (41–45).

Transcriptional regulation of miRNA levels is well illustrated by miRNAs simultaneously produced from the same genetic cluster. It is generally assumed that miRNAs located within a ca. 50 kb sequence are transcribed as polycistronic pri-miRNAs (46,47). Thus, miRNAs produced from the same cluster mostly show similar changes in their expression levels under different conditions (48). However, it has been demonstrated that the production of some miRNAs from the same polycistronic pri-miRNA can be significantly unequal due to AS, APA or the use of alternative promoters (49). Selection of alternative transcription start/termination sites (TSSs/TTSs) can be used to express distinct sets of miRNAs from a single genetic cluster and is developmentally regulated by different signalling pathways (50).

Engagement of MBNL proteins in the regulation of miRNA expression has been reported sparsely; for one miRNA, miR-1, it has been shown that MBNL1 binding to the terminal loop of its pre-miRNA precursor enhances cleavage by Dicer (51). On the other hand, it has been demonstrated that loss of MBNL1 activity is not responsible for altered expression of miRNAs in the hearts of DM1 patients and a mouse model (52). Nevertheless, comprehensive studies describing the involvement of MBNLs in regulating miRNAs in skeletal muscles, major tissues affected in DM, are missing. To address this lack, we have performed deep sequencing of the miRNA pool (microtranscriptome) of skeletal muscles of animal models with genetic knockout of *Mbnl*s and in the DM1 mouse model, *HSA*-LR, expressing mutant RNA containing expanded CUG repeats. We identified miRNAs sensitive to MBNL depletion and described the mechanisms underlying some changes in microtranscriptome composition *via* regulation of pri-miRNA splicing. Finally, we compared these results with microtranscriptome data for skeletal muscle biopsies of healthy individuals and DM1 patients and found many similarities in miRNA expression changes compared to those studied in DM1 mouse models. Moreover, we linked expression alterations of MBNLs during postnatal development of skeletal muscles with miRNA level changes occurring during this process. The results reveal a group of miRNAs potentially regulated by MBNLs. Our data are in line with studies identifying MBNL1 as a critical regulator of muscle differentiation through transcriptome-wide control of RNA metabolism.

## Materials and methods

### Genetic construct preparation

The pEGFP-MBNL-41 expression vector and *Atp2a1*ΔΔ splicing minigenes (Atp2a1-BS and Atp2a1-ΔBS) have been previously described (53,54) Atp2a1ΔΔ_wt, Atp2a1ΔΔ_mir23b_WT and Atp2a1ΔΔ_mir23b_mut plasmids were prepared based on a previously described protocol (53,55). All of the DNA sequences were purchased from oligo.pl® (Institute of Biochemistry and Biophysics PAN, Warsaw, Poland). To obtain the DNA sequences of interest (mir23b_WT, mir23b_mut), two strands of ss DNA were duplexed initially at 95°C for 1 min and at room temperature for the next 60 min. A fter digestion with respective restriction enzymes, the obtained DNA sequences containing unique restriction sites for NotI and SalI, were ligated into the Atp2a1Δ minigene and transformed into DH5α bacterial cells.

### Cell culture and transfection

MEFs_WT and MEFs*_Mbnl1&2KO* were gifts from Maurice Swanson. HeLa cells (ATCC), MEFs_WT and MEFs*_Mbnl1&2KO* were grown in high-glucose DMEM (Lonza) supplemented with 10% fetal bovine serum (FBS) (Sigma) and 1x penicillin−streptomycin (Sigma Aldrich). LHCN-M2 cells (normal human myoblasts) were purchased from Evercyte. Myoblast cells were grown in HAM F-10 medium (Lonza) supplemented with 20% FBS (BioWest), 2 mM l-glutamine, 0.055 μg/ml Dexamethasone (Sigma-Aldrich), EGF (Milteny iBiotec), 100 U/ml penicillin and 100 μg/ml streptomycin (Invitrogen). The cells were grown at 37°C in an atmosphere containing 5% CO_2_. Prior to transfection, the cells were plated in 6-well or 12-well plates and transfected at 50–60% confluence with plasmids using Lipofectamine 3000 (Thermo Fisher Scientific) or with siRNA or antisense oligonucleotides using Lipofectamine RNAiMAX (Thermo Fisher Scientific) according to the manufacturer's protocol. Genes were knocked down with siRNAs against MBNL1, MBNL2, DGCR8 and Drosha (or siCTRL was administered) at 12.5 nM or 50 nM. The siRNAs used in this study were synthetized by FutureSynthesis (siMBNL1, siMBNL2 and siCTRL) or ordered from Dharmacon (SMARTpool siDGCR8, siDrosha and siMbnl2). The cells were harvested 48 h after transfection. The siRNA sequences are listed in Supplementary Table S10. For transfection with genetic constructs, eGFP-MBNL1 and Atp2a1ΔΔ minigenes, HeLa cells were plated on the appropriate cell culture vessels. Cells were transfected 24 h after plating with genetic constructs at ∼80% confluency.

### Mouse tissue collection and RNA extraction

This study received ethical approval from the National Ethics Committee for Animal Testing. All animal procedures and endpoints were in accordance with the ARRIVE guidelines, and animals were sacrificed in accordance with the National Ethics Committee for Animal Testing and Polish guidelines and regulations. CO2 inhalation was utilized for euthanasia in accordance with Directive 2010/63/EU. The mice were housed under specific pathogen-free conditions in the animal facility of the Center of Advanced Technologies, Adam Mickiewicz University, Poznan, Poland. This is a type of animal house maintained in the SPF category. Muscle tissues from WT, *Mbnl1*KO, *Mbnl2*KO and *HSA*-LR mice were gifts from Maurice Swanson. Three (for each time point) C57BL/6J mice were sacrificed at embryonic day 18.5 and postnatal days 1, 5, 14 and 90, after which RNA was extracted from skeletal muscles (quadriceps) in TRI reagent (Sigma) using a bead-based homogenizer (TissueLyser II, Qiagen) (2 × 45 s, max frequency) and 1.4-mm and 2.8-mm ceramic beads (Qiagen). Rapid RNA purification was performed using a Total RNA Zol-out kit (A&A Biotechnology) including DNase treatment according to the manufacturer's protocol.

### DM1 patient sample characterization

The RNA samples from muscle tissues of DM1 patients were provided by Charles Thornton. The samples were obtained from the quadriceps muscles. Subjects with DM1 were ambulatory adults with proven CTG expansions, although the precise number of repeats was not determined. The initial DM1 sample was obtained from a 50-year-old female subject who exhibited the symptoms at the age of 43. The second sample was procured from a 44-year-old female subject who manifested the symptoms at the age of 29, and the third sample was taken from a 55-year-old male subject who displayed the symptoms at the age of 47. The non-DM samples were obtained from two females, aged 35 and 52, respectively.

### Splicing analyses of mature mRNA

RNA from HeLa cells and MEFs was prepared in TRI reagent (Sigma) and then subjected to rapid purification using a Total RNA Zol-out kit (A&A Biotechnology) including DNase treatment according to the manufacturer's protocol. Total RNA (1–2 μg) was reverse-transcribed using hexamers and a TranScriba Kit (A&A Biotechnology) according to the manufacturer's protocol. Splicing analyses of the MBNL1 targets were conducted with the primers listed in Supplementary Table S10. All PCR experiments were conducted with GoTaq Flexi DNA polymerase (Promega). PCR products were resolved on agarose gels with ethidium bromide (Sigma Aldrich) and visualized on a G:BOX (Syngene). Signal analyses were performed using GneTools software. To analyze splicing, the percent spliced-in (PSI) value and ΔPSI, which represents the difference in PSI between the control and tested conditions, were calculated. The majority of the tested splicing events were relatively short, which resulted in a reduction in amplification bias between splice isoforms with alternative exon inclusion or exclusion.

### Analyses of RNA levels

Total RNA was extracted from cells with TRIzol reagent (Invitrogen) according to the manufacturer's protocol. Complementary DNA (cDNA) was prepared from 1 μg of total RNA using a GoScript Reverse Transcription System (Promega). cDNA equivalent to 10 ng of the initial RNA input was used as a template for quantitative PCR (qPCR). For miRNA analysis, cDNA was synthesized in a coupled polyadenylation reverse transcription reaction by using 2 μg of total RNA for 1 h at 37°C in RT buffer (10 mM Tris–HCl pH 8.0, 75 mM KCl, 10 mM DTT, 70 mM MgCl2, 20 U RNasin and 2.5 mM of all four deoxynucleoside triphosphates; 0.5 mM of rATP, and 800 ng of anchored oligo (dT) primer) supplemented with 200 U of Superscript III reverse transcriptase (Invitrogen) and 5 U of *Escherichia coli* poly (A) polymerase (PAP, New England Biolabs)). The reactions were heat-inactivated for 10 min at 85°C. Then, 2 μl of the 9 × diluted cDNA template was used for each qPCR with a reverse primer complementary to the anchored sequence and a probe-specific forward primer (600 nM each)(56). RT−qPCR was performed using Power SYBR Green PCR Master Mix (Applied Biosystems, USA), and samples were run in technical triplicates on a 7900HT Fast Real-Time PCR instrument. The Ct values were normalized against those of the internal control: GAPDH, U6 or 5S rRNA. Fold differences in expression levels were calculated according to the 2^−ΔΔCt^ method. The primers are listed in Supplementary Table S9. Assays to quantify mature miRNAs were performed using a TaqMan MicroRNA Reverse Transcription Kit (Applied Biosystems, Foster City, CA, USA) and an RT-primer containing miRNA-specific stem−loop primers for miR-1, miR-133a, miR-133b, miR-206 and U6 (Thermo Fisher assay-IDs: 002222, 002246, 002247, 000510, 000391 and 001973). qPCR was conducted with Maxima SYBR Green Rox (Thermo Fisher Scientific) on a QuantStudio 7 Flex instrument (Thermo Fisher).

### Small RNA sequencing and data analysis

For small RNA sequencing, 200 ng of total RNA was used to prepare libraries with a NEXTflex Small RNA-Seq Kit (Bioo Scientific). The sequencing parameters were as follows: 1 × 50 bp, NovaSeq 6000. When analysing miRNA changes in *Mbnl1*KO, *Mbnl2*KO and DM1 patients, small RNA-seq data were subjected to adapter clipping with an in-house Python script, and redundant reads were removed with a fastx_collapser tool from the FASTX-Toolkit package (http://hannonlab.cshl.edu/fastx_toolkit/). The reads in FASTA format were then mapped against a set of mouse or human pre-miRNA sequences obtained from the miRBase 21 database 42 using Megablast from the BLAST package, version 2.2.26 43. It was required that the reads mapped in a sense orientation with no mismatches over the full read length. Then, using an in-house Python script, miRNA expression levels were calculated in the following way: raw counts of all reads mapped to a pre-miRNA sequence in a region occupied by an annotated mature miRNA ± 2 nt were summed. The raw expression values served as input for subsequent differential expression analysis with DESeq2 using default settings with a threshold of an adjusted *P* value <0.05. To analyze modifications of small RNA fragments, only adapter-containing reads with a length of at least 12 nt were kept. Then, the reads were filtered for quality with fastq_quality_filter from the FASTX-Toolkit with the criterion that a minimum of 95% of nucleotides had a Phred quality score of at least 20. Finally, redundant reads were removed and reads were converted to the FASTA format with fastx_collapser from the same package. The processed reads were then mapped against mouse or human pre-miRNA sequences downloaded from the miRBase database using Bowtie, with allowance of up to 2 mismatches (-v 2 option) and a requirement that all found alignments be reported (-a). Using the mapped read data and known mouse or human mature miRNA sequences from miRBase, miRNA modifications were identified with miRMOD (57) using the following settings: (i) the read count threshold was set to 2 from a default of 10; (ii) identification of modifications at both mature miRNA ends was selected; and (iii) both trimming and nucleotide addition modifications were selected.

For analyses of miRNA changes in the mouse developmental panel, reads with lengths between 10 and 35 nt were selected with cutadapt (v1.18) for downstream analysis. Raw reads were aligned to the *Mus musculus* genome (GRCm38 downloaded from the Ensembl database) with Bowtie2 (v2.4.1) (58). The mapped reads were counted with featureCounts (v2.0.1)(59) using the *Mus musculus* miRNA annotation file from mirBase, release version 22 (60). The differential expression of miRNAs was analyzed with the DESeq2 (v1.30.1) package using the likelihood ratio test (LRT) (2014, Genome Biology) in R. The miRNA expression patterns in a time course were detected with the DEGreport (v1.26.0) package in R. To find isoforms of miRNA, size-selected reads were used. FASTQ files were converted to FASTA files and collapsed with the fastx-toolkit (v0.0.13) FASTQ-to-FASTA converter and FASTQ/A Collapser, respectively. The reads were mapped to pre-miRNAs (downloaded from mirBase, release 22) with Bowtie. Isoforms of miRNA were identified with miRMOD using aligned reads from the previous step and a FASTA file with mature miRNA sequences obtained from miRbase release 22.

### Statistical analysis

The statistical significance of two independent groups was determined by an unpaired Student's *t*-test. The variances across the means of different groups were compared by ANOVA.; ∗ for *P* < 0.05, ∗∗ for *P* < 0.01 and ∗∗∗ for *P* < 0.001. All quantification of miRNA or gene expression levels was based on at least three experimental replicas and was performed with GraphPad Prism 8.3.0 (https://www.graphpad.com/). The statistical analysis for linear regression was performed using the GraphPad Prism 8.3.0 tool.

## Results

### Loss of MBNL1 and MBNL2 in skeletal muscles affects the expression of numerous miRNAs

**Figure 1.**
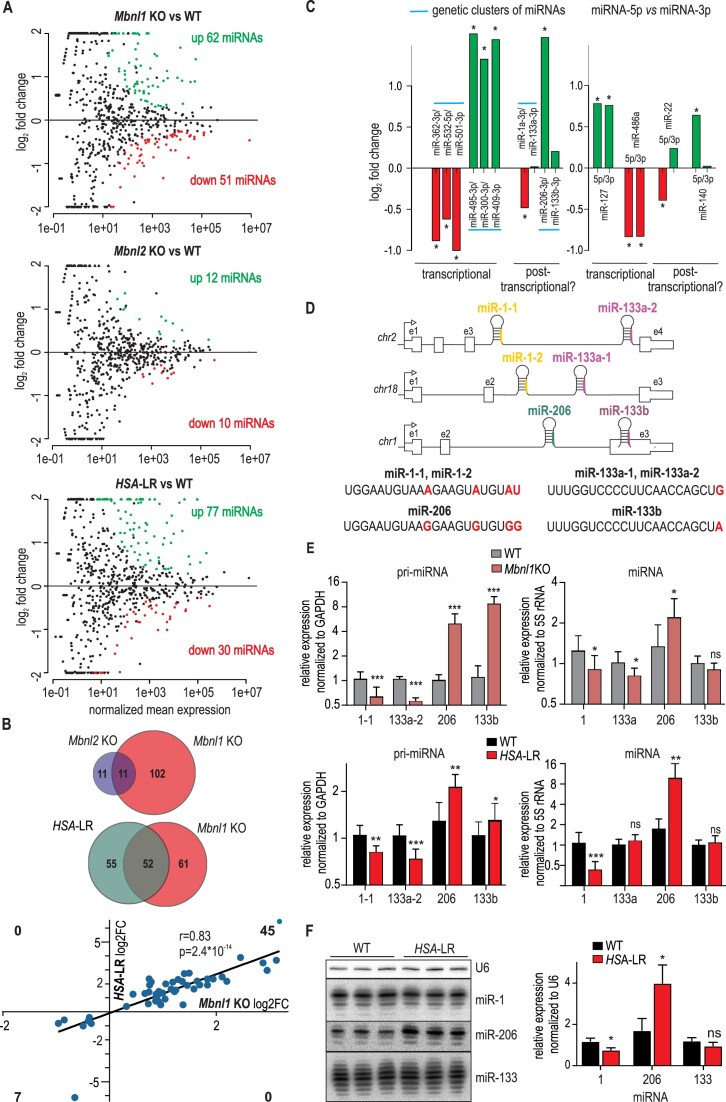
miRNA expression changes in skeletal muscles of mice with genetic knockout or MBNL1 or MBNL2 insufficiency. (**A**) MA plots indicating the dysregulated miRNA species in the muscles of *Mbnl*1KO, *Mbnl*2KO and *HSA-*LR mice compared with the control subjects. Statistically significant (*P*_adj_ < 0.05) species are represented by red dots (downregulated miRNAs) and green dots (upregulated miRNAs). (**B**) The upper Venn diagram depicts the proportion of differentially expressed miRNAs in mice with loss of Mbnl1 or Mbnl2; half of miRNAs deregulated in *Mbnl2*KO mice were also deregulated in *Mbnl1*KO mice (the only exceptions were miRNAs that were not expressed in *Mbnl1*KO mice above the cutoff of a baseMean > 10). The lower Venn diagram shows the number of miRNAs differentially expressed in *Mbnl1*KO and *HSA-*LR mice. There is a 50% overlap in miRNA changes between *Mbnl1*KO and *HSA-*LR mice. The graph shows the correlation of miRNA changes in Mbnl1KO and *HSA*-LR. The direction of all significant miRNA changes is the same in both models. Pearson correlation coefficient (*r*) and *P* value are indicated. (**C**) Expression changes of miRNAs coming from the same pre-miRNA hairpin or from the same genetic cluster (marked with blue lines) based on RNA-seq data. MiRNA candidates that could be regulated at the transcript level or posttranscriptionally are indicated. (**D**) Schematic layout of miR-1/-133a and miR-206/-133b genetic clusters and differences in sequences of miRNAs (marked in red). (**E**) Real-time PCR results for the levels of pri-myomiRNAs and mature myomiRNAs in *Mbnl1*KO and *HSA-*LR mice; all data for pri-miRNA are averages from three independent experiments ± SDs normalized to the mRNA level of GAPDH; the data for miRNA were normalized to the 5S rRNA level (* *P* < 0.05, *** *P* < 0.001, unpaired *t*-test). (**F**) Results of northern blotting of RNA extracted from *HSA*-LR mice and WT control animals; quantification of miRNAs is depicted in the graph, and the results were normalized to those of U6 (*P< 0.05, unpaired *t*-test).

To assess the global impact of MBNLs on the skeletal muscle microtranscriptome, we first sequenced a small RNA pool isolated from the quadriceps of 12-week-old mice with *Mbnl1* knockout (*Mbnl1*KO) and *Mbnl2* knockout (*Mbnl2*KO) and littermate wild-type mice (WT) (double knockout of *Mbnl*s is lethal) (*Mbnl1*dE3/dE3 mice (61); *Mbnl2*dE2/dE2 (13). Plain miRNA counts were identified from the small RNA-seq input samples and mapped against all known mouse miRNA precursor sequences deposited in miRBase. Over 450 mature miRNAs were identified (cutoff of a baseMean > 10; the mean of normalized counts of all samples, normalizing for a sequencing depth greater than 10), among which 113 were predicted to be differentially expressed (*P*_adj_ < 0.05; 90 differentially expressed if *P*_adj_ < 0.01) between WT and *Mbnl1*KO mice (Supplementary Table S1). Approximately equal numbers of miRNAs were up- and downregulated (62 and 51, respectively), indicating that the regulatory impact of *Mbnl1*KO on miRNA levels is not limited to activation or inhibition (Figure 1A). Comparing WT and *Mbnl2*KO, we identified only 22 differentially expressed miRNAs (Figure 1A and Supplementary Table S2). Changes in miRNA levels in the muscles of *Mbnl1*KO mice were deeper and more abundant than those in the *Mbnl2*KO mice; however, many miRNAs deregulated in the *Mbnl2*KO mice were also deregulated in the *Mbnl1*KO mice (Figure 1B and Supplementary Table S3). Therefore, we concluded that similar to the case in AS regulation, MBNL1 plays a prevailing role in miRNA governance in skeletal muscles, as its expression is highest among the MBNL paralogs in this tissue. Thus, in further studies, we focused on miRNAs misregulated in *Mbnl1*KO. Interestingly, among the most deregulated miRNAs, we found crucial muscle-specific miRNAs, including miR-1, miR-206, and miR-486. MiR-206 was upregulated, and miR-1 was substantially downregulated. Gene Ontology (GO) analysis (62) revealed that 113 miRNAs misregulated in *Mbnl1*KO mice are associated with cellular component organization and cell differentiation, especially differentiation of muscle tissue (Supplementary Figure S1a). As many as 97 genes involved in muscle development were identified as targets of deregulated miRNAs. Analyzing previously published results of microarray-based gene expression changes in skeletal muscles of *Mbnl1*KO mice (63), we found that the expression of 31 of these genes (40% of 78 expressed genes) was significantly deregulated (*P*_adj_ < 0.05) (Supplementary Table S4), which indicates the significant effect of miRNA dysregulation on gene expression in this animal model.

A similar analysis, using the same pipeline, has been carried out for the *HSA*-LR mouse (Supplementary Table S5). *HSA*-LR mice express the transgene with 220 CTG repeats in the 3′UTR and reproduce some symptoms noticed in patients, such as myotonic discharges, myopathy and splicing defects. In this model, all MBNL paralogs are sequestered on CUG^exp^ in nuclear foci, and loss of their function accounts for DM-specific missplicing (64,65). Overall, the *HSA*-LR mouse can be regarded, in many ways, as a model for functional knockdown of MBNLs. By monitoring miRNA changes in this model, we found almost 50% overlap in miRNA changes between *Mbnl1*KO and *HSA*-LR mice (Figure 1B). This value increases to 60% when using a cut-off of log2FC > 0.5. Furthermore, the direction of all miRNA changes in Mbnl1KO and *HSA*-LR mice is the same (significantly changed in both models, cut-off of a baseMean > 10) (Figure 1B). Thus, most of the cellular processes perturbed by deregulated miRNAs overlap in both mouse models (Supplementary Figure S1a).

*Mbnl1*KO mice display overt myotonia and muscle dystrophy beginning at approximately 6 weeks of age. To determine whether the microtranscriptome changes are specific to MBNL1 deficiency or are a consequence of muscle pathology (e.g. myotonia or muscle dystrophy), we assessed whether similar miRNA changes were observed in a mouse model of facioscapulohumeral muscular dystrophy (FSHD). FSHD manifests as myotonic disorders resulting from rare genetic mutations affecting skeletal muscle function. We compared publicly available data of miRNA changes in muscles of the FSHD mouse model based on DUX4 expression (66) and in *Mbnl*1KO mice. As expected, some miRNA changes were shared between the *Mbnl1*KO and FSHD mouse models (e.g. miR-206). However, the deregulation of some other miRNAs involved in muscle development, including miR-1, miR-486 and miR-27b, was not recapitulated in this model (Supplementary Figure S1b), suggesting that microtranscriptome changes observed in *Mbnl1*KO are not due to impaired skeletal muscle function per se.

To estimate whether miRNA changes observed in *Mbnl1*KO depend on transcriptional or posttranscriptional processes, we analyzed all deregulated miRNAs, accounting for their origin from either the same miRNA genetic clusters or the same pre-mRNA (two miRNAs can be produced from the two arms of pre-miRNA: 5p and 3p) (67). Most miRNAs misregulated in *Mbnl1*KO mice belonged to miRNA clusters, of which many members had altered levels in the same direction (Figure 1C), which suggested that they were deregulated at the transcript level. Similarly, concordant dysregulations were observed for many miRNA pairs, 5p and 3p, coming from the same pre-miRNA. Based on these assumptions, we determined that 56 out of 90 differentially expressed miRNAs were at least partially deregulated at the transcript level (Supplementary Table S6). On the other hand, for some muscle-specific miRNAs that belonged to the same genetic cluster, we did not observe such a correlation. For example, significant downregulation of muscle-specific miR-1 (*P*adj = 0.0014) was not accompanied by a change in miR-133a, and upregulation of miR-206 (*P*_adj_ = 4.78E–36) was not followed by a significant increase in miR-133b (Figure 1C). To reveal whether these discrepancies were a result of the use of independent TSSs for each pri-miRNA within genetic clusters, we examined the levels of pri-miRNAs in the direct vicinity of each miRNA. There are two polycistronically transcribed pri-miRNA precursors of miR-1/133a: miR-1-1/133a-2 and miR-1–2/133a-1 (Figure 1D). We found that pri-miR-1-1/133a-2 was expressed at a much higher level and that its level was decreased in *Mbnl1*KO mice (Figure 1E). On the other hand, pri-miR-206/133b was significantly upregulated when measured near one or the other miRNA. The accordant alterations at the level of pri-miRNA and subsequent discrepancies at the level of mature miRNA indicated that (i) pri-miR-1-1/133a-2 and pri-miR-206/133b are transcribed together, and abnormalities in transcription are at least partially responsible for differences in the levels of mature miRNAs, and (ii) additional posttranscriptional correction of miR-133a and -133b levels exists.

We also confirmed decreased levels of miR-1 and increased levels of miR-206 but the levels of the pool of miR-133s in skeletal muscle in *HSA*-LR, the DM1 mouse model, was unchanged (Figure 1E). To check whether the quantitative changes were not accompanied by different distributions of the length variants of these miRNAs, we visualized them by Northern blotting (Figure 1F). In this model, as in *Mbnl1*KO mice, the levels of pri-miR-1-1/133a-2 and pri-miR-206/133b were down- and upregulated, respectively (Figure 1E).

Previously, the MBNL-dependent regulation of miR-1 expression at the posttranscriptional level was described. It was proposed that in the heart MBNL1 binds to the terminal loop of the pre-miR-1 hairpin structure and enhances its processing by the ribonuclease DICER (51). However, the decreases in pri-miR-1-1 in the skeletal muscle of *Mbnl1*KO and *HSA*-LR mice observed in this study suggest that reduced levels of mature miR-1 could also be a consequence of deregulation at the transcript level. Thus, we decided to evaluate whether posttranscriptional deregulation takes place upon MBNL deficiency for two miR-1 and miR-133a precursor variants and miR-206 and miR-133b precursors. We generated constructs for overexpression of these miRNA precursors. To test whether they are directly regulated by MBNLs, we delivered them into HeLa cells with or without *MBNL1*&*2* knockdown or to cells overexpressing MBNL1. We did not observe any significant differences in the levels of the studied miRNAs (Supplementary Figure S1c and d, the efficacy of MBNL knockdown and overexpression is demonstrated in Supplementary Figure S1e). Therefore, we concluded that in HeLa cells, there are no direct, meaningful effects on the levels of the studied muscle-specific miRNAs upon MBNL knockdown.

### *Mbnl1* knockout affects the expression of a subset of miRNAs developmentally regulated in skeletal muscles

**Figure 2.**
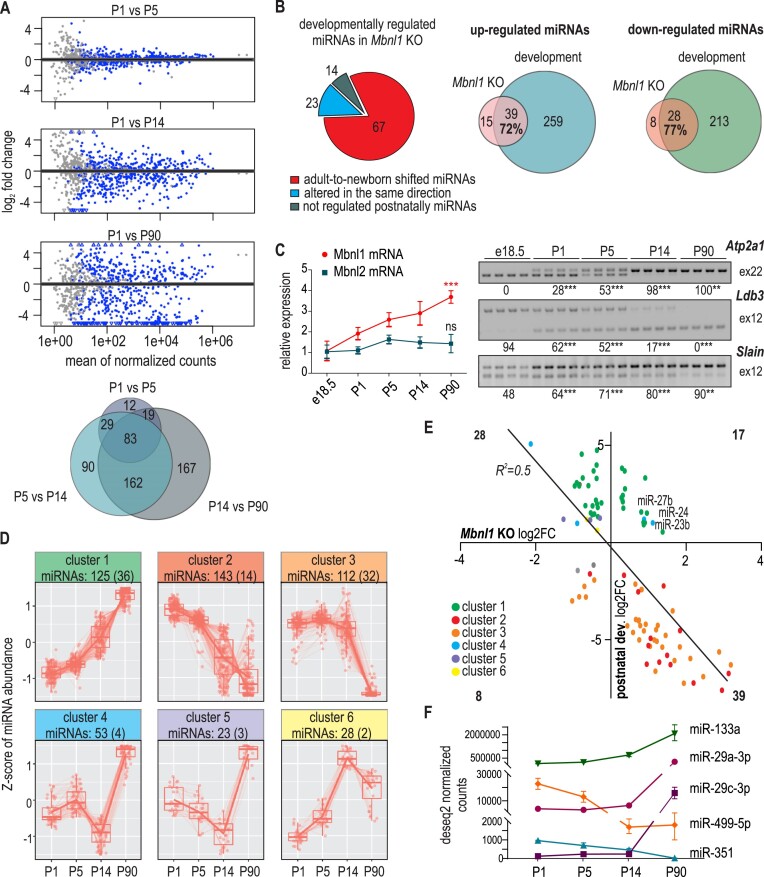
Comparison of miRNA expression changes during mouse postnatal skeletal muscle development and in *Mbnl1*KO mice. (**A**) MA plots showing the predicted differentially expressed miRNAs muscles between P1 and P5 and between P14 and P90. The experiment was performed for the quadriceps of WT mice in triplicate. The Venn diagram shows the number of miRNAs whose levels changed in mouse muscles between three different time points of postnatal development. (**B**) Pie chart summarizing changes in developmentally regulated miRNAs in *Mbnl1*KO; two Venn diagrams with developmentally regulated miRNAs and those regulated in *Mbnl1*KO mice are shown (http://www.biovenn.nl/index.php was used to generate the diagram). (**C**) *Mbnl1* and *Mbnl2* gene expression during the development of skeletal muscle (*n* = 3) as determined by RT-qPCR expression analyses. All data are averages from three independent experiments ± SDs normalized to the mRNA expression of GAPDH (* *P* < 0.05, ** *P* < 0.01, *** *P* < 0.001). Representative gels and calculations of RT−PCR analyses of AS events regulated by MBNLs during development. The data represent the mean PSI values ± SDs (*n* ≥ 3). Statistical significance was calculated in reference to the control (E18.5) using two-way Anova; ns, nonsignificant, ∗ for *P* < 0.05, ∗∗ for *P* < 0.01, ∗∗∗ for *P* < 0.001. (**D**) Plots showing six clusters of miRNA expression profiles during postnatal development. MiRNAs were classified based on the course of their expression changes; the number of miRNAs within the cluster is indicated. In the brackets, the number of miRNAs deregulated in *Mbnl1*KO mice is depicted. The degPatterns function from the ‘DEGreport’ package was used to determine sets of genes that exhibit similar expression patterns across sample groups. (**E**) Correlation between miRNAs that were significantly changed in *Mbnl1*KO mice and those that were significantly changed during development (P90 versus P1) according to small RNA-seq data. The dot colors indicate the six developmental clusters to which the miRNAs belong (classified as in D). The graph shows a negative correlation between miRNA expression changes in *Mbnl1*KO mice and miRNA expression changes during development. The strength of a linear correlation and its statistical significance were determined by Pearson's correlation coefficient. (**F**) Pattern of expression of selected miRNAs during postnatal development of skeletal muscles. The data are shown for miRNAs whose levels were altered significantly during development but not upon MBNL1 knockout based on RNA-seq experiments.

It is well established that in skeletal muscles, MBNL1 participates in the postnatal remodeling of the transcriptome of protein-coding genes (12,68,69). Accordingly, *Mbnl1*KO mice display several molecular and physiological abnormalities in this tissue type. To determine how many miRNAs that were deregulated in *Mbnl1*KO are engaged in muscle development, we sequenced the microtranscriptomes of mouse quadriceps from different developmental stages, postnatal days 1 (P1), 5 (P5), 14 (P14) and 90 (P90; adults). Differentially expressed miRNAs at subsequent time points were visualized by MA plots (Figure 2A). The miRNA expression changes that occurred between P1 and P5, P5 and P14, and P14 and P90 were determined. Over time, the number of differentially expressed miRNAs increased. Eighty-three miRNAs were found to be differentially expressed in both young (P5 and P14) and adult mice (P90) compared to newborns (P1) (*P*_adj_ < 0.05); however, there were many miRNAs that were differentially expressed only in a specific temporal window of muscle development (Figure 2A; Venn diagram). A comparison of miRNAs that were changed in *Mbnl1*KO mice and during postnatal development (P90 versus P1) revealed that as many as 90 out of 104 miRNAs with abnormal levels in *Mbnl1*KO mice (87%) were regulated during normal postnatal muscle development. The majority of these miRNAs (67/90; 75%) were negatively correlated for the compared groups and exhibited some adult-to-newborn shift in *Mbnl1*KO mice (Figure 2B). On the other hand, the majority of miRNAs showing differential expression in the developmental process were not affected in *Mbnl1*KO mice (88%). We concluded that deficiency of MBNL1 resulted in disruption of a small subset of developmentally regulated miRNAs. However, based on this comparison, it was not possible to distinguish between miRNA changes induced directly by MBNL1 insufficiency and those induced by secondary effects caused by impairment of muscle development.

Then, we investigated the correlation between the dynamics of MBNL1 expression changes and those of miRNA changes during muscle development, which might further indicate the dependence of miRNA on MBNL1. Between P1 and P90, we observed a gradual increase in the level of *Mbnl1* mRNA, while the level of *Mbnl2* mRNA increased only between P1 and P5 (Figure 2C left graph; Supplementary Figure S2c). We monitored the postnatal splicing switch for three exons known to be MBNL1-regulated since it has been demonstrated previously that in mice, the transition from inclusion to exclusion of MBNL-dependent alternative exons occurs mainly after birth between postnatal days 2 and 16 (10). We found that similar to changes in the *Mbnl1* mRNA level, the inclusion rate of all tested exons changed continuously throughout development (Figure 2C; right). In the next step, we analyzed the miRNA-seq data from the postnatal muscle development panel in the context of MBNL1-sensitive miRNAs. According to the dynamics of microtranscriptome changes, we divided miRNAs into six groups (Figure 2D). Assuming that MBNL1-sensitive miRNAs identified in *Mbnl1*KO mice would follow the MBNL1 activity pattern, we concluded that they would be included mostly in clusters 1 and 2, characterized by gradual increases and decreases in miRNA levels, respectively. As many as 36 out of 125 miRNAs from cluster 1 (29%) and 14 out of 143 from cluster 2 (10%) were misregulated in *Mbnl1*KO mice (Figure 2D and Supplementary Table S7). Notably, the vast majority of miRNAs that changed upon postnatal muscle development were not disrupted in *Mbnl1*KO mice (ca. 88%). Among miRNAs disturbed in *Mbnl1*KO mice, we did not find miRNAs that substantially changed upon muscle development, i.e. miR-29a (log2FC = 5.9, *P*_adj_ = 6.6e-117), miR-29c (log_2_FC = 6.9, *P*_adj_ = 1.0e-160), miR-351 (log_2_FC=-5.7, *P*_adj_ = 2.3e-51), or, surprisingly, muscle-specific miR-133a (log2FC = 3.7, *P*adj = 2.2e-67) and miR-499 (log_2_FC = 6.0, *P*_adj_ = 2.2e-07) (Figure 2E, F). The selectivity of miRNA changes observed in *Mbnl1*KO mice suggested that full developmental reprogramming of the microtranscriptome did not occur with a clear transition from the adult pattern to earlier developmental stages.

Knowing that MBNLs influence miRNA quantity in the muscles of adult mice, we decided to examine whether they also affect microtranscriptome quality by analyzing the distribution of miRNA sequence variants in *Mbnl1*KO mice. IsomiRs are miRNA isoforms resulting from RNA modifications mediated by specific enzymes and differing in length and/or sequence from the canonical forms. Small differences in the length and sequence of mature miRNAs are responsible for their specificity, stability and effectiveness (70). Sequencing of the microtranscriptome has shown that isomiRs are differentially expressed during development and in various tissues (71,72). To determine potential changes in the distribution of isomiRs in *Mbnl1*KO mice and during muscle development, we used the miRMod program, which enables identification of 5′- and 3′-sequence modifications (57). We did not observe any global alterations in 3′- or 5′-nucleotide additions or 5′-trimming between control and *Mbnl1*KO muscles; the only change was a slight increase in 3′-trimming (Supplementary Figure S2a, b). On the other hand, we observed significant changes in the distribution of all types of these modifications between developmental stages, mostly in the second (P14) and twelfth weeks (P90) (Supplementary Figure S2d). The most prominent were differences in oligouridylation and oligoadenylation. Since the oligouridylation decrease is very steep during muscle development (ca. 3-fold), the lack of change in U-tailing in *Mbnl1*KO mice is another argument in favor of the lack of a general adult-to-newborn shift in miRNA expression in this mouse model.

### MiRNAs from the miR-23b/27b/24-1 genetic cluster are dysregulated in DM mouse models

**Figure 3.**
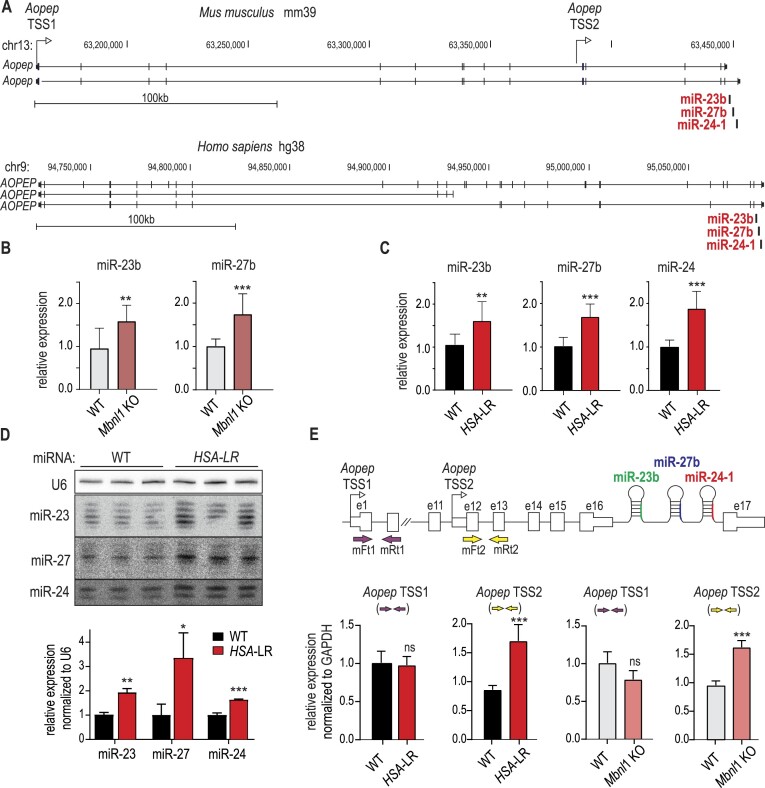
Expression changes of miRNAs coming from genetic clusters located within the mouse and human *AOPEP* genes. (**A**) Schematic gene outline based on UCSC genome browser visualization of the mouse (GRCm39/mm39) and human genomes (GRCh38/hg38) (https://genome.ucsc.edu). In the last intron of both *Aopep* genes, the locations of the miR-23b, miR-27b, and miR-24-1 sequences are indicated. (**B**) Validation of miR-23b and miR-27b levels in *Mbnl1*KO and WT animals by RT−qPCR. All data are averages from three independent experiments ± SDs normalized to the levels of U6 (* *P* < 0.05, ** *P* < 0.01, *** *P* < 0.001, unpaired t-test). (**C**) The same as in B but for *HSA*-LR mice. (**D**) Northern blot analysis of total RNA extracted from *HSA*-LR mice and WT control animals and its quantification. This method did not enable discrimination between miR-23a and miR-23b or miR-27a and miR-27b but did enable quantification of miRNA isoforms differing in length. The quantification of miRNAs is depicted in the graph, and the results were normalized to those of U6 (*P< 0.05, unpaired *t*-test). (**E**) Quantitative analysis of RNA expression for two regions of the *Aopep* gene in *Mbnl1*KO and *HSA*-LR mice. For both mouse models, the level of RNA in the region proximal to the miRNA sequence was elevated (*Aopep* RNA from TSS2; amplified fragment of exons 12 and 13 with the primer pair indicated with yellow arrows), while the level of RNA for the distal fragment of *Aopep*, located upstream of the potential alternative TSS (TSS2), was not significantly altered in either *Mbnl1*KO or *HSA*-LR mice (*Aopep* RNA from TSS1 amplified with primers indicated with purple arrows). The data are averages from three independent experiments ± SDs normalized to *Gapdh* levels (* *P* < 0.05, ** *P* < 0.01, *** *P* < 0.001, unpaired *t*-test).

In further studies, we wanted to look deeper into the potential mechanisms of miRNA regulation by MBNLs. Knowing that part of the miRNA misregulation in *Mbnl1*KO mice can be a secondary effect of muscle abnormalities, we identified a group of miRNAs whose levels were altered during postnatal development and in *Mbnl1*KO mice in the same direction (Figure 2E). Among the 16 members of this group, six miRNAs belonged to two genetic clusters: miR-23a/27a/24-2 and miR-23b/27b/24-1. MiRNAs produced from the miR-23b/27b/24-1 cluster were some of the most significantly changed in *Mbnl1*KO mice and were highly expressed in skeletal muscles. Their sequences were localized in an approximately 800 bp fragment of the last intron of the *Aopep* gene in both humans and mice (Figure 3A).

Using miRNA-specific RT−qPCR assays, we confirmed substantial increases in miR-23b and miR-27b levels in *Mbnl1*KO mice (Figure 3B) and showed the same increases in *HSA*-LR mice (Figure 3C). Moreover, these quantitative changes were not accompanied by different distributions of length variants of these miRNAs visualized by northern blotting (Figure 3D). The simultaneous increases in the levels of all three miRNAs from the cluster suggested a transcriptional mechanism of deregulation in *Mbnl1*KO and *HSA*-LR mice. To validate this hypothesis, we analyzed several fragments of pri-miRNAs 23b/27b/24-1. We detected increases in the RNA levels of *Aopep* in regions flanking all three miRNA sequences but not in regions that included upstream sequences transcribed presumably from TSS1 of *Aopep* in both *Mbnl1*KO and *HSA-*LR mice (Figure 3E). We concluded that the activity of alternative TSSs of *Aopep* can be a source of dysregulation of miR-23b, miR-27b and miR-24 in DM mice (Figure 3E).

### MBNLs regulate AS of the miR-23b/miR-27b/miR-24-1 primary transcript

**Figure 4.**
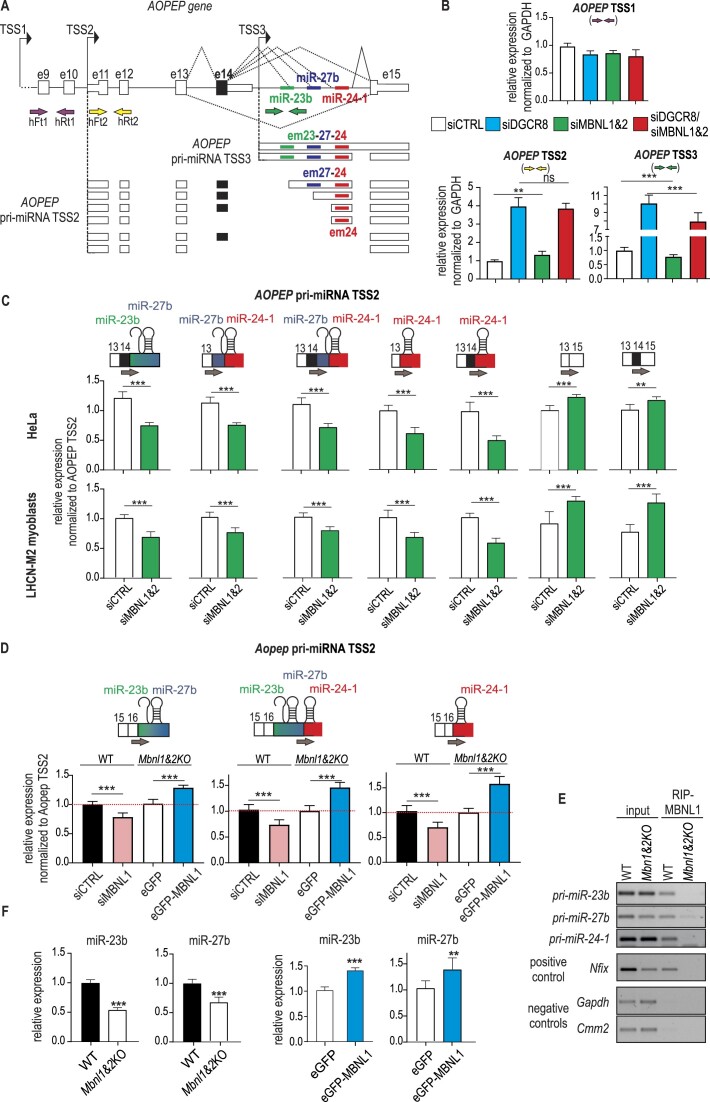
Pri-miRNAs of miR-23b, miR-27b and miR-24-1 are alternatively spliced and are sensitive to MBNL levels. (**A**) Splicing isoforms of two populations of pri-miRNAs produced from two TSSs, TSS2 and TSS3, of the *AOPEP* gene identified in this study. Exons encoding mRNA isoforms (e11–e15) and exons encoding pri-miRNA isoforms (em24, em27 and em24) are marked. The locations of the three pre-miRNA sequences are indicated with different colors. The locations of the primers used in the experiments are indicated with arrows. (**B**) RT−qPCR-based expression analyses of the transcripts generated from three alternative TSSs, TSS1, TSS2 and TSS3. All data are mean values from three independent experiments ± SDs normalised to GAPDH (* *P* < 0.05, ** *P* < 0.01, *** *P* < 0.001, unpaired t-test). (**C**) RT−qPCR-based expression analyses of the pri-miRNAs in HeLa and LHCN-M2 cells upon siMBNL1&2 treatment. In each assay, the discriminating forward primer was anchored at the junction of the AOPEP constitutive exon and the alternatively spliced exon with the miRNA sequence (the aforementioned primer is presented on the scheme above the graph). All data are averages from three independent experiments ± SDs normalized to AOPEP TSS2 mRNA expression in order to eliminate the possibility that observed changes are biased by whole transcriptional unit expression (* *P* < 0.05, ** *P* < 0.01, *** *P* < 0.001, unpaired t-test). (**D**) RT−qPCR-based expression analyses of the pri-miRNAs in MEF WT and *Mbnl1&2*KO cells upon siMBNL1 or eGFE-MBNL1 treatment. In each assay, the discriminating forward primer was anchored at the junction of the AOPEP constitutive exon and the alternatively spliced exon with the miRNA sequence (the aforementioned primer is presented on the scheme above the graph). All data are averages from three independent experiments ± SDs to AOPEP TSS2 mRNA expression in order to eliminate the possibility that observed changes are biased by whole transcriptional unit expression (* *P* < 0.05, ** *P* < 0.01, *** *P* < 0.001, unpaired t-test). (**E**) RIP enrichment analysis was performed using an MBNL1-specific antibody on the WT or *Mbnl1*&*2*KO (1&2KO) MEFs. RT-PCR was performed for RNA isolated from inputs and MBNL1 immunoprecipitates (RIP-MBNL1) using primers flanking each of the pri-miRNA fragments. The positive control in this experiment was mRNA of *Nfix*, which is a target of MBNL1 (60), and the negative controls were the mRNAs of *Gapdh* and *Cmm2*. (**F**) The alterations in the levels of miR-23b and miR-27b in *Mbnl1&2*KO cells in comparison to WT MEFs are presented on the first two graphs. The third and fourth graphs demonstrate the restoration of miR-23b and miR-27b expression in *Mbnl1&2*KO MEFs upon the exogenous overexpression of GFP-MBNL1 or GFP. The results were estimated by RT−qPCR. The level of miR-23b was normalized to that of U6 (*P < 0.05, **P < 0.01, ***P < 0.001, unpaired *t*-test).

To gain insight into the mechanism of miR-23b, miR-27b, and miR-24-1 biogenesis, we first aimed to identify pri-miRNA sequences from which these miRNAs are generated. We designed a few sets of primers that enabled analysis of RNA species arising from the host human and mouse *AOPEP* gene and its last intron (Supplementary Figure S3a, S5a) using HeLa cells and mouse embryonic fibroblasts (MEFs), respectively. As transcripts that are primary precursors of miRNAs are short-lived due to fast processing by the DROSHA/DGCR8 microprocessor complex, we knocked down members of this complex in the tested cells using siRNA. Using this approach, we identified several different human pri-miRNA variants as substrates for the microprocessor. In humans, there were at least five alternatively spliced variants produced from two TSSs (TSS2 and TSS3). The first three consisted of sequences between exons 11 and 15 of *AOPEP* (TSS2) and included alternative exons containing a sequence of either two pre-miRNAs, pre-miR-27b and pre-miR-24, or just one, pre-miR-24-1 (Figure 4A and Supplementary Figures S3b,e and S4). Two other transcripts produced from alternative TSS3 contained sequences of all three pre-miRNAs (Supplementary Figure S3c). Based on RNA ligase-mediated rapid amplification of cDNA ends (RLM 5′RACE), which amplifies cDNA only from full-length, capped mRNA, we found that the 5′-end of this second pri-miRNA was located 529 nucleotides upstream of miR-23b and did not overlap with exon 14 of *AOPEP* (Supplementary Figure S3c). Similar profiles of pri-miRNA sequences were observed in mouse MEFs (Supplementary Figure S5b). We detected four RNA isoforms that lacked consecutive pre-miRNA sequences (Figure 4A). These observations suggest that in addition to transcriptional regulation, the expression of miR-23b, miR-27b and miR-24 can also be modulated by AS.

In order to ascertain whether any of these AS events is regulated by MBNLs, a comparison was made between the levels of identified primary miRNA precursors in control HeLa cells and cells with a knockdown of MBNL1 and MBNL2 (MBNL1&2_KD). To detect alternative miRNA exons in HeLa MBNL1&2_KD with primers anchored in constitutive AOPEP exons, it was necessary to simultaneously knockdown DGCR8. In cells treated with control siRNA, only two AOPEP mRNA variants lacking miRNA sequences were identified, which exhibited a sensitivity to siMBNL1&2 (inclusion of alternative exon 14) (Supplementary Figure S3e; green bar). However, in cells also treated with siDGCR8, more differences were observed. In MBNL1&2_KD cells, the levels of exons including miR-27b and miR-24-1 were significantly decreased (Supplementary Figure S3d and e). Similar changes were observed in mouse cells (MEFs) with *Mbnl1&2*KO (Supplementary Figure S5c) in the context of siDrosha (Supplementary Figure S5d).

To confirm results obtained with semiquantitative RT-PCR and quantify mRNA isoforms, including miRNA sequences, in an RT-qPCR assay, primers were designed to span the junctions of the AOPEP exon and the newly identified alternative miRNA exon (em23b, em27b, em24-1) (Figure 4C). To eliminate bias that can be introduced by increased expression of AOPEP transcript (Figure 4B), the level of pri-miRNAs were normalized to AOPEP TSS2. The results indicated that the newly identified pri-miRNA isoforms exhibited MBNL sensitivity. In HeLa MBNL1&2_KD cells (the level of MBNL1 and MBNL2 knockdown is shown in Supplementary Figure S6a and b), the levels of exons including miR-23b, miR-24-1 and miR-27b/24-1 were significantly decreased (Figure 4B). At the same time, an increase in mRNA without alternative exons was observed. We observed also a reduction in mature miR-23b and miR-24 levels in HeLa cells following MBNL1&2 knockdown in the presence of siDGCR8 (Supplementary Figure S6c). This is despite the fact that miRNAs are considered to be relatively stable molecules with long half-lives (>20 h) (29). Subsequently, the experiment was repeated in the human myoblast cell line LHCN-M2, yielding comparable results. Furthermore, we observed a decline in the inclusion of miR-23b, miR-24-1 and miR-27b/24-1 exons in mouse cells MEFs WT upon Mbnl1 knockdown (Figure 4D).

Since all identified exons with miRNA sequences of the miR-23b/27b/24-1 cluster were regulated by MBNLs, we looked for MBNL-binding sequence motifs in the last intron of the *AOPEP* gene. We found many 5′-YGCY-3′ sequence motifs (73,74) near miR-23b and miR-27b (Supplementary Figure S7a), and based on our previously published results of MBNL-specific RIP-seq experiments (54); available at MIB.amu.edu.pl), we identified a few MBNL binding site candidates that can play a role in AS regulation. To further confirm these predictions, we performed RT-PCR on a product of RNA immunoprecipitation (RIP) performed with an antibody against MBNL1. In this experiment, we used cell extract from WT MEFs (MEFs_WT) and MEFs obtained from double-*Mbnl1&2* knockout MEFs (MEFs_*Mbnl1&2*KO) as negative controls. RNA pulldown from WT cell extract resulted in a significantly higher signal from primary transcripts for miR-23b, miR-27b, and miR-24-1 compared to that from MEF_*Mbnl1&2*KO cell extract (Figure 4E). To confirm direct binding of MBNL1 to selected RNA regions, we used the previously described *Atp2a1ΔΔ* splicing minigene system, which was designed to enable evaluation of functional MBNL-binding sites within tested RNA sequences (53). We inserted the pre-miR-23b fragment containing potential MBNL-binding sites downstream of the alternative exon of *Atp2a1*, which is positively regulated by all MBNL paralogs (Supplementary Figure S7b). Cotransfection of the construct with the MBNL1-overexpression vector resulted in significantly higher inclusion of the alternative *Atp2a1* exon. In contrast, the inclusion of the exon was unaffected when the potential MBNL-binding sequence located in the pre-miR-23b fragment was mutated (Supplementary Figure S7c). In the final experiment, we investigated whether modification of the MBNL pool affected the levels of miRNAs generated from the analyzed genetic cluster. Overexpression of MBNL1 in MEFs_*Mbnl1&2*KO resulted in a significant increase in the level of pri-miR-23b/-27b/-24-1 (Figure 4D) and consequently in mature miR-23b and miR-27b (Figure 4F).

All these results provide evidence that MBNLs play essential roles in the regulation of miRNAs from the miR-23b/miR-27b/miR-24-1 cluster. The mechanism involves positive regulation of AS of exons that include the pre-miRNA sequences promoting the production of mature miRNAs.

### The insufficiency of MBNLs contributes to microtranscriptome deregulation in the muscles of DM1 patients

**Figure 5.**
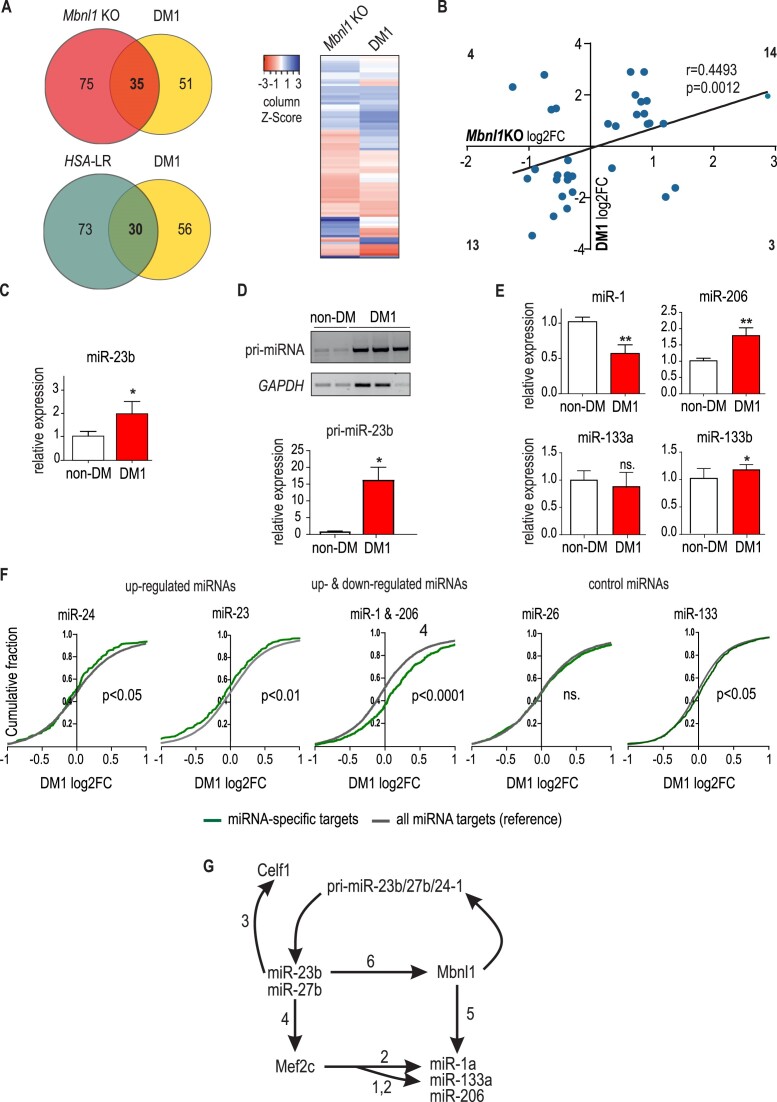
Contribution of MBNLs insufficiency to miRNA changes observed in skeletal muscles of DM1 patients. (**A**) The upper Venn diagram shows the number of miRNAs whose levels were significantly changed in the muscles of *Mbnl1*KO mice and the muscles of DM1 patients (P_adj_ < 0.05, cutoff of a baseMean > 10). Note that some of miRNAs misregulated in the mouse model were not detected in patient samples, and vice versa. The lower Venn diagram shows the number of miRNAs whose levels were significantly changed in the muscles of *HSA-LR* mice and the muscles of DM1 patients It is noteworthy that of the 103 miRNAs significantly deregulated in *HSA*-LR, 60 miRNAs were not detected in DM1 sequencing data with cutoff of a baseMean > 10. The heatmap represents the miRNA signature of MBNL deficiency. Eighty-six miRNAs significantly deregulated in *Mbnl1*KO mice and expressed in DM1 samples are shown. Positive Z scores (red) indicate enrichment in upregulated miRNAs, negative scores (blue) indicate enrichment in downregulated miRNAs, and scores centered on zero (white) mean minimal difference. (**B**) The graph presents a comparison of miRNA expression changes in two sets of experiments: *Mbnl1*KO mice vs. patients with DM1. The scatter plots of miRNA log_2_FC expression values were created by comparison of the same 35 miRNAs significantly changed in *Mbnl1*KO mice (Padj < 0.05) and in muscle biopsies of DM1 patients. Pearson's r = 0.4493 (moderate positive); *P* = 0.0012 (**). (**C**) RT−qPCR measurement of miR-23b levels in the muscles of DM1 patients normalized to those of U6 (* *P* < 0.05). (**D**) Steady-state level of pri-miR23b from the *AOPEP* cluster evaluated by RT−PCR in healthy controls, DM1 patients and normalized to the level of *GADPH*. (**E**) The levels of four myomiRs from three genetic clusters (miR-1-1/miR-133a-2, miR-1–2/miR-133a-1, miR-206/miR-133b) determined by RT−qPCR and normalized to the level of U6 (* *P* < 0.05; ** *P* < 0.001). (**F**) Cumulative distributions of gene expression changes expressed as log_2_FC for all mRNAs (black line) and mRNAs that are targets for miR-23, miR-24, miR-1&-206 (as miR-1 and miR-206 share the same targets), miR-133, and a control miR-26 (green lines). The data for the analysis come from the microarray experiment described in (75). Note that miR-24 and miR-23 targets were significantly downregulated, while targets of miR-1 and miR-206 were significantly upregulated, compared to the expression change profile of all mRNAs. (**G**) The auto-regulatory MBNL1/miR-23 loop and the involvement of myomiRs. The scheme combines previously published information and results from this manuscript. The numbers above the arrows indicate the publication from which the information is taken: 1) Liu *et al.*, Proc Natl Acad Sci U S A 2007 (106); 2) Rota *et al.*, Mol Cancer 2011 (107); 3) Kalsotra *et al.*, Genes Dev. 2010 (108); 4) Chinchilla *et al.*, Cardiovascular Research 2011 (87); 5) Rau *et al.*, Nat Struct Mol Biol. 2011 (51); 6) Cerro-Herreros *et al.*, Nat. Commun. 2018 (90).

Considering that MBNL participates in miRNA regulation, its functional insufficiency should be reflected in the microtranscriptome of skeletal muscles of DM1 patients. To profile the composition and expression of miRNAs, we isolated total RNA from muscle biopsies of DM1 (*n* = 3) and non-DM controls (*n* = 2) and performed small RNA-seq according to the same pipeline as for the mouse microtranscriptome. Among 500 identified miRNAs (cutoff of a baseMean > 10; mean of normalized counts of all samples, normalizing for a sequencing depth greater than 10), as many as 86 were changed significantly (*P*_adj_ < 0.05) (Figure 5A and Supplementary Table S8). Among them, 35 miRNAs were deregulated in both DM1 patients and the *Mbnl1*KO mouse model. Importantly, 76% of these miRNAs deregulated in *Mbnl1*KO mice exhibited changed expression in DM1 in the same direction (Figure 5B). However, the fold changes were mostly higher in patients than in the mouse model. A comparison of miRNA changes in DM1 patients and *HSA*-LR mice revealed that 30 miRNAs were deregulated in both (Figure 5A). It is noteworthy that of the 103 miRNAs significantly deregulated in *HSA*-LR, 60 miRNAs were not detected in DM1 sequencing data with cutoff of a baseMean > 10; which revealed that 30 (70%) of the 43 miRNAs that were significantly altered in *HSA*-LR and highly expressed in DM1 muscles were also deregulated in DM1 patients (Supplementary Figure S7e).

As in the two tested DM mouse models, we also observed increases in the levels of miRNAs generated from two genetic clusters, miR-23a/27a/24-2 and miR-23b/27b/24-1. For miR-23b, we validated expression changes at the level of mature miRNA and its pri-mRNA using an independent set of DM1 skeletal muscles (Figure 5C, D). Furthermore, the levels of two major myomiRs, miR-1 and miR-206, were altered in the same manner as in *Mbnl1*KO and *HSA-*LR mice, i.e. miR-1 expression was decreased and miR-206 expression was increased, while the level of miR-133a (generated from the same genetic clusters as miR-1) was unchanged, and that of miR-133b (clustered with miR-206) was only marginally upregulated (Figure 5E).

To reveal whether miRNA changes are translated to deregulation of their mRNA targets, we analyzed whole-transcriptomic data obtained for DM1 muscles, which we have previously described (75). First, we identified potential mRNA targets for several MBNL-dependent miRNAs using miRTarBase, which contains experimentally validated miRNA targets (although some miRNA:target interactions may be indirect or otherwise weak). (76). On a global scale, we observed significant downregulation of miR-23 and miR-24 targets, which was consistent with the upregulation of these miRNAs in DM1 skeletal muscles. As miR-1 and miR-206 have the same seed sequence, they share the majority of mRNA targets. We found that this group of endogenous targets of both miRNAs was selectively upregulated in DM1. This indicates a dominant impact of insufficiency of miR-1 over the increase in miR-206 expression in shaping the DM1 transcriptome (Figure 5F). We also looked more globally at the effects of microtranscriptome changes on the expression of protein-coding genes. We used miRNet version 2.0 to obtain an integrated picture of gene expression changes in muscle tissue and the miRNAs that regulate them. The results demonstrated that among all miRNAs deregulated in DM1, miR-1 had the greatest influence on disrupted gene expression (Supplementary Figure S7d).

Both mouse models that we used are the best-accepted models of DM1, and they appear well suited to assessing DM1-associated miRNA deregulation. Indeed, we observed disruption of the levels of numerous miRNAs in the muscles of DM1 patients. According to gMicroRNA ENrichment TURned NETwork (MIENTURNET) (Licursi V et al BMC Bioinformatics. 2019), the deregulated genes in DM1 muscle biopsies are predicted targets of over 60 miRNAs. Since miRNAs play critical roles in stress responses, differentiation, proliferation, and apoptosis in muscle (77), they might contribute to muscle pathobiology in DM1.

## Discussion

MBNL proteins are well-known regulators of RNA metabolism. The functional deficit of MBNLs in DM leads to impairment of RNA metabolism, specifically posttranscriptional AS and APA (1,2). In addition to quantitative and qualitative disturbances in the expression of hundreds of mRNAs, deregulation of miRNA levels is also observed in DM1 patients (24–26,78). However, the molecular basis of miRNA deregulation has not been explained by previously published data, except for that of miR-1. It has been proposed that MBNL1 binds within the terminal loop of the hairpin structure formed by pre-miR-1 and prevents its uridylation by ZCCHC11 (TUT4) driven by Lin28, which promotes Dicer processing. We hypothesized that MBNL proteins may also affect miRNA levels by regulating the levels and processing of primary miRNA precursors (pri-miRNAs). According to recent reports, more than half of human and mouse miRNAs have intragenic locations (68). Sixty-five percent of them are intronic miRNAs, while exonic miRNAs account for just 3%, and junction miRNAs account for 2.5% of all miRNAs. Splicing, as a major event in the processing of RNA Pol II transcripts, constitutes the first level of posttranscriptional regulation before pre-miRNA cleavage by the microprocessor. Cotranscriptional AS modifying specific pri-miRNA sequences may affect the biogenesis of miRNAs at the level of efficiency of pre-miRNA excision. In addition to miRNA location, the second factor that can influence miRNA levels, is the interaction of proteins with miRNA precursors. MBNLs can operate within both regulatory pathways.

To reveal whether MBNL proteins have an impact on the microtranscriptome of skeletal muscles, we analyzed small RNA fractions isolated from skeletal muscles of mouse models with a deficiency of MBNL1 or MBNL2 as well as in *HSA-*LR, the DM1 mouse model with sequestration of all MBNLs. We observed significant deregulation of miRNA levels in *Mbnl1*KO mice, whereas in *Mbnl2*KO mice, we detected only minor changes. This may indicate that similar to the predominant splicing activity of MBNL1 over MBNL2 in skeletal muscles (3,54,79), the same preference exists for miRNA biogenesis. Moreover, similar changes in miRNA expression were observed in *Mbnl1*KO and *HSA-*LR mice. Reports on MBNL1 protein levels during the postnatal development of mouse muscle have been inconsistent; authors have noted an Mbnl1 increase (80) or decrease (10). Therefore, we decided to examine Mbnl1 expression in our muscle developmental panel, and we detected its upregulation at both the mRNA and protein levels (81). Analysing the splicing activity of Mbnl1 during postnatal growth of muscle confirmed the increase in Mbnl1 and the continuous involvement of Mbnl1 in splicing regulation throughout the examined period of development. We revealed that MBNL1 is involved in secondary myogenesis not only because of splicing and mRNA level regulation but also because it triggers changes in the miRNA level.

*Mbnl1*KO mice display several muscle abnormalities, including overt myotonia beginning at approximately 6 weeks of age that is caused by abnormal AS of *Clcn1* mRNA, an increase in mislocalized nuclei, and splitting of myofibers. It is also known that MBNL1 participates in postnatal skeletal muscle development (12,69), as its level significantly increases during this process. Therefore, the greatest challenge in analyzing the factors involved in muscle development is distinguishing between changes derived directly from factor deficiency itself and those that are indirect consequences of impaired or retarded myogenesis. Undoubtedly, the miRNA deregulation observed in muscle models with MBNL1 deficiency is the effect of both. Nevertheless, in the Mbnl1KO mice, we did not observe a significant disruption in the levels of developmentally regulated miRNAs (Figure 2B; right), making it unlikely that the observed miRNA changes were the result of development retardation alone. Notably, adult WT mice and *Mbnl1*KO mice were age matched (∼12 weeks old). The different stabilities of individual miRNAs could explain the selectivity of the observed alterations; however, we belive that depletion of MBNLs specifically impacts a subset of miRNAs involved in muscle development, leaving the remaining developmentally related miRNAs unaffected. Another argument in favor of MBNL1-triggered miRNA changes was that *Mbnl1*KO mice lack isomiR disturbances, including profound decreases in uridylation and adenylation (Supplementary Figure S2a). We revealed that significant changes in miRNA variant levels occurred during postnatal muscle remodeling (Supplementary Figure S2d); thus, muscle growth retardation would have resulted in isomiR composition alterations in *Mbnl1*KO mice. The abundance of isomiRs varies between cell types (82) and during cell transitions (83,84); however, there are no reports characterizing the dynamics of miRNA modification during tissue development. Our data indicate that muscle differentiation is linked to vast changes in miRNA variant distribution, but further studies are needed to extend our findings using improved miRNA tail-seq methods and a wider range of myogenesis stages in order to reveal the importance of miRNA modifications for muscle differentiation.

MyomiRs are a subset of miRNAs shown to be muscle-enriched that play key roles in myogenesis and muscle function (85). In muscle microtranscriptome analyses, their levels inevitably draw attention. Interestingly, only a decrease in miR-1 expression and an increase in miR-206 expression were observed in *Mbnl1*KO mice. Neither the level of miR-133a clustered with miR-1 nor the level of miR-133b clustered with miR-206 was altered. MiR-1 and miR-133a are supposed to be derived from the same pri-miRNA polycistronic transcript (86). Physiologically, throughout the early postnatal period and mouse adolescence, the endogenous levels of miR-1 and miR-133a increase significantly before reaching a plateau in the adult muscles (87). However, some reports have suggested that miR-1 can also be regulated independently from miR-133a (51,88). Previously, it has been shown that MBNL1 directly regulates the excision of the miR-1 duplex from its pre-miRNA; however, our data also indicated that miR-1 and miR-206 change at the transcript level, with clear pri-miRNA level alterations in two MBNL1-deficient mouse models (Figure 1E and F) and in DM1 patients (Figure 5). What remains to be explained in future studies is how miR-133a and miR-133b expression is autonomously controlled in these conditions, as the pri-miRNA level changes were not followed by alterations in mature miRNA-133a or -133b levels.

We also demonstrated that miR-23b, miR-27b, and miR-24, which belong to the same genetic cluster, were among the most significantly and consistently changed in the muscles of mouse models with MBNL deficiency (*Mbnl1*KO and *HSA-*LR). They were particularly interesting to us since their changes could not be attributed to muscle development-dependent retardation. Previous studies have shown that members of the miR-23b/-27b/-24-1 cluster play important roles during cardiac and skeletal muscle development by controlling some crucial muscle drivers, including *Mef2c* (89) and *Pax3* (90). Furthermore, miR-23b has been described as a direct repressor of *MBNL1* and *MBNL2* (91–93), which might imply the existence of a regulatory feedback loop (Figure 5G). Our study showed that MBNL1 is involved directly in the regulation of miR-23b levels through several lines of evidence. First, the knockdown of MBNL1 resulted in a decrease in the pri-miR-23b level and significant changes in the AS pattern of this precursor, leading to deletion of the pre-miR-23b-containing sequence (Figure 4B). Second, overexpression of MBNL1 in MEF_*Mbnl1&2*KO cells resulted in a significant increase in miR-23b (Figure 4F). Third, a direct interaction between the pri-miR-23b fragment and MBNL1 was confirmed in RIP experiments and using an MBNL-sensitive splicing minigene containing the miR-23b flanking region with MBNL-binding sites (Figure 4E and Supplementary Figure S7c).

The regulation mechanism of miRNA biogenesis from the miR-23b/-27b/-24-1 cluster seemed to be complex. We could not determine whether three miRNAs (miR-23b, miR-27b and miR-24-1) could be generated from the same pri-miR-23b/27b/24-1 molecule or whether two miRNAs (miR-27b and miR-24-1) could arise from pri-miR-27b/24-1. If only one pre-miRNA could be cut out from a single primary transcript, the elimination of individual pre-miRNA sequences from pri-mRNA would provide an intriguing mechanism that controls miRNA biogenesis. Cutting out the introns with pre-miRNA sequences would result in several different pri-miRNA isoforms (Figure 4A), from which one, two, or all three pre-miRNAs could be generated by the microprocessor. In fact, it was shown that from the miR-23a/27a/24-2 cluster the most miR-24 was produced when construct included only miR-24 sequence, less when the miR-27a/24 construct was used and the least was generated from the miR-23a/27a/24 construct (94). Splicing-dependent regulation of intronic clusters has been previously reported in several publications. Melamed *et al.* found that a splicing event not only splits the miRNA cluster (miR-541, miR-409, miR-412 and miR-369) but also specifically hinders the processing of miR-412 (95). In addition, splice isoforms that do not belong to the host gene isoforms have been shown to be produced exclusively to uncouple cluster miRNA expression (96–98). However, elucidation of miR-23b, miR-27b and miR-24 generation was beyond the scope of this work, so further studies are necessary to understand the process.

Notwithstanding the foregoing, the splicing mechanism seemed to overlap with the regulation of *Aopep* expression. Since all four detected mouse pri-miRNA isoforms consisted of *Aopep* exons plus additional exons with miRNA sequences, it was assumed that their expression changed along with the *Aopep* transcript level and that the final amounts of miRNAs generated from the miR-23b/-27b/-24-1 cluster depended both on the transcription rate of pri-miRNAs and their AS regulated by MBNL1. Additionally, TSSs (TSS1, TSS2 and TSS3) could be used to express distinct *Aopep* RNA isoforms. Indeed, in *Mbnl1*KO and *HSA-*LR mice, we observed increases in *Aopep* RNA levels in regions flanking pre-miRNA sequences and a lack of change in the distant part of *Aopep* mRNA (Figure 3E). Therefore, we hypothesized that the increases in the levels of miRNAs from the miR-23b/ -27b/ -24-1 cluster in the muscles of *Mbnl1*KO and *HSA-*LR mice resulted from transcriptional enhancement of the TSS2 transcription unit, which masked the opposite effects of changes in AS we observed in different cellular models. The observed upregulation of *Aopep* TSS2 may result from epigenetic alterations or disruption of transcription factors expression induced, most likely indirectly, by MBNL1 deficiency. In conclusion, MBNL1 has been shown to positively regulate the expression of miR-23b, -27b and -24-1 via direct involvement in the alternative splicing of the pri-miRNA, and to exert an indirect effect on the level of the Aopep TSS2 transcript.

The analysis of miRNAs levels in our study, besides smallRNA-seq, is based on RT-qPCR assays, which remain the most popular technique used to date. However, the ability of RT-qPCR approaches to distinguish between 3′-isomiRs or between miRNA family members that differ by a single nucleotide can be poor if not carefully designed (99). Nevertheless, the polyadenylation-based RT-qPCR method has been demonstrated to be capable of selective amplification of individual miRNA isoforms or effective discrimination between miRNAs from the same family when designed appropriately (100). Consequently, the primers utilized for miR-23a and miR-23b, miR-27a and miR-27b, and miR-133a and miR-133b were designed in such a manner that the discriminating nucleotide was the last nucleotide at the 3′ end of the primer. Although non-complementarity can be tolerated at the internal or 5′ nucleotides of the primer, the requirement for complementarity appears to be strong for the 3′ base (101). To assess the discriminatory power of the used primers, we have analyzed the CT values obtained for miRNAs belonging to the same family in the same sample.

Our findings suggest that functional insufficiency of MBNL proteins can be partially responsible for altered miRNA expression in DM1 patients. The significant portion of convergent changes in miRNA levels observed in the skeletal muscles of mice with *MBNL*s knockout and in the muscles of DM1 patients indicates that MBNL functional knockdown leaves an imprint on the DM1 microtranscriptome and, consequently, on the expression of hundreds of protein-coding genes (Figure 5A, F). Dysfunction of miRNA expression is hypothesized to be the basis of many different pathologies, including DM1 (102), to some extent, but it is difficult to separate overlapping primary and secondary effects of deregulating numerous miRNAs and ascertain which element of disease they are responsible for. Nevertheless, the analysis of our previously published whole-transcriptome data from skeletal muscles of DM1 patients enabled us to create an interaction network of gene expression changes and miRNAs that are potentially responsible for them. This analysis showed that the change with the highest impact on perturbed mRNA steady-state levels in DM1 was a reduced level of miR-1 (Supplementary Figure S7d). This was somewhat expected, as miR-1 is the miRNA with the highest expression in skeletal muscles and as even a small change in its level significantly disrupts the stoichiometry of miR-1 molecules and their individual targets. miR-1 and miR-206 were semihomologous, with similar mature sequences and identical seed sequences. They had some independent targets; however, most of them were shared. Although the miR-1 level was decreased and the miR-206 level was increased in DM1, the levels of targeted transcripts were elevated, indicating the predominant role of miR-1 in their regulation. This observation is consistent with reports of the broad impacts of decreases in miR-1 levels on muscle physiology (103–105). The impact of reduced miR‐1 expression on DM1 pathogenesis has been shown recently in the heart of a *Drosophila* DM1 model (104). The authors observed that the *dmiR‐1* level was reduced in the cardiac cells of DM1 flies and that its downregulation in the heart led to dilated cardiomyopathy (DCM), thus suggesting that reduced *dmiR‐1* levels contribute to DM1‐associated DCM.

In summary, our data support the hypothesis that decreased activity of MBNLs results in changes in numerous miRNAs. Most of them are regulated during postnatal skeletal muscle development, and the adult-to-newborn shift in expression of these miRNAs is observed in mouse models of DM and in DM1 patients. We hypothesized that Mbnl1 deficiency influences miRNA levels through a combination of mechanisms, including through regulation of AS of primary miRNAs precursors.

## Supplementary Material

gkae774_Supplemental_Files

## Data Availability

The RNA-seq data are available at NCBI GEO under GSE245818,GSE268937 and GSE268938 accession numbers. All the relevant data underlying this article are available in its online supplementary material and archived in Zenodo with the following DOIs: 10.5281/zenodo.13477050.

## Notes

Present address: Michał Sekrecki, Hematology Division, Department of Medicine, Stanford University School of Medicine, 269 Campus Dr Rm 1155 MC 5156 Stanford, CA 94305, USA.

## References

[1] Pascual M. , Vicente M., Monferrer L., Artero R. The Muscleblind family of proteins: an emerging class of regulators of developmentally programmed alternative splicing. Differentiation. 2006; 74:65–80. 16533306 10.1111/j.1432-0436.2006.00060.x

[2] Batra R. , Charizanis K., Manchanda M., Mohan A., Li M., Finn D.J., Goodwin M., Zhang C., Sobczak K., Thornton C.A. et al. Loss of MBNL leads to disruption of developmentally regulated alternative polyadenylation in RNA-mediated disease. Mol. Cell. 2014; 56:311–322. 25263597 10.1016/j.molcel.2014.08.027 PMC4224598

[3] Wang E.T. , Cody N.A.L., Jog S., Biancolella M., Wang T.T., Treacy D.J., Luo S., Schroth G.P., Housman D.E., Reddy S. et al. Transcriptome-wide regulation of pre-mRNA splicing and mRNA localization by muscleblind proteins. Cell. 2012; 150:710–724. 22901804 10.1016/j.cell.2012.06.041 PMC3428802

[4] Masuda A. , Andersen H.S., Doktor T.K., Okamoto T., Ito M., Andresen B.S., Ohno K. CUGBP1 and MBNL1 preferentially bind to 3’ UTRs and facilitate mRNA decay. Sci. Rep. 2012; 2:209. 22355723 10.1038/srep00209 PMC3250574

[5] Lee J.E. , Cooper T.A. Pathogenic mechanisms of myotonic dystrophy. Biochem. Soc. Trans. 2009; 37:1281–1286. 19909263 10.1042/BST0371281 PMC3873089

[6] Timchenko L. Molecular mechanisms of muscle atrophy in myotonic dystrophies. Int. J. Biochem. Cell Biol. 2013; 45:2280–2287. 23796888 10.1016/j.biocel.2013.06.010 PMC3759660

[7] Hildebrandt R.P. , Moss K.R., Janusz-Kaminska A., Knudson L.A., Denes L.T., Saxena T., Boggupalli D.P., Li Z., Lin K., Bassell G.J. et al. Muscleblind-like proteins use modular domains to localize RNAs by riding kinesins and docking to membranes. Nat. Commun. 2023; 14:3427. 37296096 10.1038/s41467-023-38923-6 PMC10256740

[8] Terenzi F. , Ladd A.N. Conserved developmental alternative splicing of muscleblind-like (MBNL) transcripts regulates MBNL localization and activity. RNA Biol. 2010; 7:43–55. 20009516 10.4161/rna.7.1.10401

[9] Kino Y. , Washizu C., Kurosawa M., Oma Y., Hattori N., Ishiura S., Nukina N. Nuclear localization of MBNL1: splicing-mediated autoregulation and repression of repeat-derived aberrant proteins. Hum. Mol. Genet. 2015; 24:740–756. 25274774 10.1093/hmg/ddu492

[10] Lin X. , Miller J.W., Mankodi A., Kanadia R.N., Yuan Y., Moxley R.T., Swanson M.S., Thornton C.A. Failure of MBNL1-dependent post-natal splicing transitions in myotonic dystrophy. Hum. Mol. Genet. 2006; 15:2087–2097. 16717059 10.1093/hmg/ddl132

[11] Kalsotra A. , Xiao X., Ward A.J., Castle J.C., Johnson J.M., Burge C.B., Cooper T.A. A postnatal switch of CELF and MBNL proteins reprograms alternative splicing in the developing heart. Proc. Natl. Acad. Sci. U.S.A. 2008; 105:20333–20338. 19075228 10.1073/pnas.0809045105 PMC2629332

[12] Brinegar A.E. , Xia Z., Loehr J.A., Li W., Rodney G.G., Cooper T.A. Extensive alternative splicing transitions during postnatal skeletal muscle development are required for calcium handling functions. eLife. 2017; 6:e27192. 28826478 10.7554/eLife.27192 PMC5577920

[13] Charizanis K. , Lee K.-Y., Batra R., Goodwin M., Zhang C., Yuan Y., Shiue L., Cline M., Scotti M.M., Xia G. et al. Muscleblind-like 2-mediated alternative splicing in the developing brain and dysregulation in myotonic dystrophy. Neuron. 2012; 75:437–450. 22884328 10.1016/j.neuron.2012.05.029 PMC3418517

[14] Fardaei M. Three proteins, MBNL, MBLL and MBXL, co-localize in vivo with nuclear foci of expanded-repeat transcripts in DM1 and DM2 cells. Hum. Mol. Genet. 2002; 11:805–814. 11929853 10.1093/hmg/11.7.805

[15] Spruce T. , Plass M., Gohr A., Ray D., Martínez de Lagrán M., Rot G., Nóvoa A., Burguera D., Permanyer J., Miret M. et al. The X-linked splicing regulator MBNL3 has been co-opted to restrict placental growth in eutherians. PLoS Biol. 2022; 20:e3001615. 35476669 10.1371/journal.pbio.3001615 PMC9084524

[16] Mahadevan M. , Tsilfidis C., Sabourin L., Shutler G., Amemiya C., Jansen G., Neville C., Narang M., Barceló J., O’Hoy K. et al. Myotonic dystrophy mutation: an unstable CTG repeat in the 3′ untranslated region of the gene. Science. 1992; 255:1253–1255. 1546325 10.1126/science.1546325

[17] Taneja K.L. , McCurrach M., Schalling M., Housman D., Singer R.H. Foci of trinucleotide repeat transcripts in nuclei of myotonic dystrophy cells and tissues. J. Cell Biol. 1995; 128:995–1002. 7896884 10.1083/jcb.128.6.995 PMC2120416

[18] Wheeler T.M. , Lueck J.D., Swanson M.S., Dirksen R.T., Thornton C.A. Correction of ClC-1 splicing eliminates chloride channelopathy and myotonia in mouse models of myotonic dystrophy. J. Clin. Invest. 2007; 117:3952–3957. 18008009 10.1172/JCI33355 PMC2075481

[19] Thomas J.D. , Oliveira R., Sznajder Ł.J., Swanson M.S. Myotonic dystrophy and developmental regulation of RNA processing. Comprehensive Physiology. 2018; Wiley 509–553. 10.1002/cphy.c170002 PMC11323716 29687899

[20] Dixon D.M. , Choi J., El-Ghazali A., Park S.Y., Roos K.P., Jordan M.C., Fishbein M.C., Comai L., Reddy S. Loss of muscleblind-like 1 results in cardiac pathology and persistence of embryonic splice isoforms. Sci. Rep. 2015; 5:9042. 25761764 10.1038/srep09042 PMC4356957

[21] Lee K.-Y. , Seah C., Li C., Chen Y.-F., Chen C.-Y., Wu C.-I., Liao P.-C., Shyu Y.-C., Olafson H.R., McKee K.K. et al. Mice lacking MBNL1 and MBNL2 exhibit sudden cardiac death and molecular signatures recapitulating myotonic dystrophy. Hum. Mol. Genet. 2022; 31:3144–3160. 35567413 10.1093/hmg/ddac108 PMC9476621

[22] Day J.W. , Ranum L.P.W. RNA pathogenesis of the myotonic dystrophies. Neuromuscul. Disord. 2005; 15:5–16. 15639115 10.1016/j.nmd.2004.09.012

[23] Misra C. , Lin F., Kalsotra A. Deregulation of RNA metabolism in microsatellite expansion diseases. Adv Neurobiol. 2018; 20:213–238. 29916021 10.1007/978-3-319-89689-2_8 PMC6323645

[24] Gambardella S. , Rinaldi F., Lepore S.M., Viola A., Loro E., Angelini C., Vergani L., Novelli G., Botta A. Overexpression of microRNA-206 in the skeletal muscle from myotonic dystrophy type 1 patients. J. Transl. Med. 2010; 8:48. 20487562 10.1186/1479-5876-8-48 PMC2880982

[25] Perbellini R. , Greco S., Sarra-Ferraris G., Cardani R., Capogrossi M.C., Meola G., Martelli F. Dysregulation and cellular mislocalization of specific miRNAs in myotonic dystrophy type 1. Neuromuscul. Disord. 2011; 21:81–88. 21169019 10.1016/j.nmd.2010.11.012

[26] Fernandez-Costa J.M. , Garcia-Lopez A., Zuñiga S., Fernandez-Pedrosa V., Felipo-Benavent A., Mata M., Jaka O., Aiastui A., Hernandez-Torres F., Aguado B. et al. Expanded CTG repeats trigger miRNA alterations in Drosophila that are conserved in myotonic dystrophy type 1 patients. Hum. Mol. Genet. 2013; 22:704–716. 23139243 10.1093/hmg/dds478

[27] Perfetti A. , Greco S., Cardani R., Fossati B., Cuomo G., Valaperta R., Ambrogi F., Cortese A., Botta A., Mignarri A. et al. Validation of plasma microRNAs as biomarkers for myotonic dystrophy type 1. Sci. Rep. 2016; 6:38174. 27905532 10.1038/srep38174 PMC5131283

[28] Ha M. , Kim V.N. Regulation of microRNA biogenesis. Nat. Rev. Mol. Cell Biol. 2014; 15:509–524. 25027649 10.1038/nrm3838

[29] Marzi M.J. , Ghini F., Cerruti B., de Pretis S., Bonetti P., Giacomelli C., Gorski M.M., Kress T., Pelizzola M., Muller H. et al. Degradation dynamics of microRNAs revealed by a novel pulse-chase approach. Genome Res. 2016; 26:554–565. 26821571 10.1101/gr.198788.115 PMC4817778

[30] Kingston E.R. , Bartel D.P. Global analyses of the dynamics of mammalian microRNA metabolism. Genome Res. 2019; 29:1777–1790. 31519739 10.1101/gr.251421.119 PMC6836734

[31] Reichholf B. , Herzog V.A., Fasching N., Manzenreither R.A., Sowemimo I., Ameres S.L. Time-resolved small RNA sequencing unravels the molecular principles of MicroRNA homeostasis. Mol. Cell. 2019; 75:756–768. 31350118 10.1016/j.molcel.2019.06.018 PMC6713562

[32] Newman M.A. , Thomson J.M., Hammond S.M. Lin-28 interaction with the let-7 precursor loop mediates regulated microRNA processing. RNA. 2008; 14:1539–1549. 18566191 10.1261/rna.1155108 PMC2491462

[33] Rybak A. , Fuchs H., Smirnova L., Brandt C., Pohl E.E., Nitsch R., Wulczyn F.G. A feedback loop comprising lin-28 and let-7 controls pre-let-7 maturation during neural stem-cell commitment. Nat. Cell Biol. 2008; 10:987–993. 18604195 10.1038/ncb1759

[34] Heo I. , Joo C., Cho J., Ha M., Han J., Kim V.N. Lin28 Mediates the terminal uridylation of let-7 precursor MicroRNA. Mol. Cell. 2008; 32:276–284. 18951094 10.1016/j.molcel.2008.09.014

[35] Viswanathan S.R. , Daley G.Q., Gregory R.I. Selective blockade of MicroRNA processing by Lin28. Science. 2008; 320:97–100. 18292307 10.1126/science.1154040 PMC3368499

[36] Davis B.N. , Hilyard A.C., Lagna G., Hata A. SMAD proteins control DROSHA-mediated microRNA maturation. Nature. 2008; 454:56–61. 18548003 10.1038/nature07086 PMC2653422

[37] Suzuki H.I. , Yamagata K., Sugimoto K., Iwamoto T., Kato S., Miyazono K. Modulation of microRNA processing by p53. Nature. 2009; 460:529–533. 19626115 10.1038/nature08199

[38] Trabucchi M. , Briata P., Garcia-Mayoral M., Haase A.D., Filipowicz W., Ramos A., Gherzi R., Rosenfeld M.G. The RNA-binding protein KSRP promotes the biogenesis of a subset of microRNAs. Nature. 2009; 459:1010–1014. 19458619 10.1038/nature08025 PMC2768332

[39] Guil S. , Cáceres J.F. The multifunctional RNA-binding protein hnRNP A1 is required for processing of miR-18a. Nat. Struct. Mol. Biol. 2007; 14:591–596. 17558416 10.1038/nsmb1250

[40] Treiber T. , Treiber N., Plessmann U., Harlander S., Daiß J.-L., Eichner N., Lehmann G., Schall K., Urlaub H., Meister G. A compendium of RNA-binding proteins that regulate MicroRNA biogenesis. Mol. Cell. 2017; 66:270–284. 28431233 10.1016/j.molcel.2017.03.014

[41] Tang X. , Zhang Y., Tucker L., Ramratnam B. Phosphorylation of the RNase III enzyme drosha at Serine300 or Serine302 is required for its nuclear localization. Nucleic Acids Res. 2010; 38:6610–6619. 20554852 10.1093/nar/gkq547 PMC2965249

[42] Paroo Z. , Ye X., Chen S., Liu Q. Phosphorylation of the Human MicroRNA-generating complex mediates MAPK/erk signaling. Cell. 2009; 139:112–122. 19804757 10.1016/j.cell.2009.06.044 PMC2760040

[43] Herbert K.M. , Pimienta G., DeGregorio S.J., Alexandrov A., Steitz J.A. Phosphorylation of DGCR8 increases its intracellular stability and induces a progrowth miRNA profile. Cell Rep. 2013; 5:1070–1081. 24239349 10.1016/j.celrep.2013.10.017 PMC3892995

[44] Wan G. , Zhang X., Langley R.R., Liu Y., Hu X., Han C., Peng G., Ellis L.M., Jones S.N., Lu X. DNA-damage-induced nuclear export of precursor MicroRNAs is regulated by the ATM-AKT pathway. Cell Rep. 2013; 3:2100–2112. 23791529 10.1016/j.celrep.2013.05.038 PMC3796289

[45] Wada T. , Kikuchi J., Furukawa Y. Histone deacetylase 1 enhances microRNA processing via deacetylation of DGCR8. EMBO Rep. 2012; 13:142–149. 22222205 10.1038/embor.2011.247 PMC3271337

[46] BASKERVILLE S. , BARTEL D.P. Microarray profiling of microRNAs reveals frequent coexpression with neighboring miRNAs and host genes. RNA. 2005; 11:241–247. 15701730 10.1261/rna.7240905 PMC1370713

[47] Liang Y. , Ridzon D., Wong L., Chen C. Characterization of microRNA expression profiles in normal human tissues. Bmc Genomics [Electronic Resource]. 2007; 8:166. 17565689 10.1186/1471-2164-8-166 PMC1904203

[48] Zhang X. , Azhar G., Wei J.Y. The expression of microRNA and microRNA clusters in the aging heart. PLoS One. 2012; 7:e34688. 22529925 10.1371/journal.pone.0034688 PMC3329493

[49] Chang T.-C. , Pertea M., Lee S., Salzberg S.L., Mendell J.T. Genome-wide annotation of microRNA primary transcript structures reveals novel regulatory mechanisms. Genome Res. 2015; 25:1401–1409. 26290535 10.1101/gr.193607.115 PMC4561498

[50] Zhou L. , Lim M.Y.T., Kaur P., Saj A., Bortolamiol-Becet D., Gopal V., Tolwinski N., Tucker-Kellogg G., Okamura K. Importance of miRNA stability and alternative primary miRNA isoforms in gene regulation during Drosophila development. eLife. 2018; 7:e38389. 30024380 10.7554/eLife.38389 PMC6066331

[51] Rau F. , Freyermuth F., Fugier C., Villemin J.-P., Fischer M.-C., Jost B., Dembele D., Gourdon G., Nicole A., Duboc D. et al. Misregulation of miR-1 processing is associated with heart defects in myotonic dystrophy. Nat. Struct. Mol. Biol. 2011; 18:840–845. 21685920 10.1038/nsmb.2067

[52] Kalsotra A. , Singh R.K., Gurha P., Ward A.J., Creighton C.J., Cooper T.A. The Mef2 transcription network is disrupted in myotonic dystrophy heart tissue, dramatically altering miRNA and mRNA expression. Cell Rep. 2014; 6:336–345. 24412363 10.1016/j.celrep.2013.12.025 PMC3927417

[53] Cywoniuk P. , Taylor K., Sznajder Ł.J., Sobczak K. Hybrid splicing minigene and antisense oligonucleotides as efficient tools to determine functional protein/RNA interactions. Sci. Rep. 2017; 7:17587. 29242583 10.1038/s41598-017-17816-x PMC5730568

[54] Sznajder Ł.J. , Michalak M., Taylor K., Cywoniuk P., Kabza M., Wojtkowiak-Szlachcic A., Matłoka M., Konieczny P., Sobczak K. Mechanistic determinants of MBNL activity. Nucleic Acids Res. 2016; 44:10326–10342. 27733504 10.1093/nar/gkw915 PMC5137450

[55] Taylor K. , Piasecka A., Kajdasz A., Brzęk A., Polay Espinoza M., Bourgeois C.F., Jankowski A., Borowiak M., Raczyńska K.D., Sznajder Ł.J. et al. Modulatory role of RNA helicases in MBNL-dependent alternative splicing regulation. Cell. Mol. Life Sci. 2023; 80:335. 37882878 10.1007/s00018-023-04927-0 PMC10602967

[56] Raczynska K.D. , Ruepp M.-D., Brzek A., Reber S., Romeo V., Rindlisbacher B., Heller M., Szweykowska-Kulinska Z., Jarmolowski A., Schümperli D. FUS/TLS contributes to replication-dependent histone gene expression by interaction with U7 snRNPs and histone-specific transcription factors. Nucleic. Acids. Res. 2015; 43:9711–9728. 26250115 10.1093/nar/gkv794 PMC4787759

[57] Kaushik A. , Saraf S., Mukherjee S.K., Gupta D. miRMOD: a tool for identification and analysis of 5′ and 3′ miRNA modifications in Next Generation sequencing small RNA data. PeerJ. 2015; 3:e1332. 26623179 10.7717/peerj.1332 PMC4662591

[58] Langmead B. , Salzberg S.L. Fast gapped-read alignment with Bowtie 2. Nat. Methods. 2012; 9:357–359. 22388286 10.1038/nmeth.1923 PMC3322381

[59] Liao Y. , Smyth G.K., Shi W. featureCounts: an efficient general purpose program for assigning sequence reads to genomic features. Bioinformatics. 2014; 30:923–930. 24227677 10.1093/bioinformatics/btt656

[60] Kozomara A. , Birgaoanu M., Griffiths-Jones S. miRBase: from microRNA sequences to function. Nucleic Acids Res. 2019; 47:D155–D162. 30423142 10.1093/nar/gky1141 PMC6323917

[61] Kanadia R.N. , Johnstone K.A., Mankodi A., Lungu C., Thornton C.A., Esson D., Timmers A.M., Hauswirth W.W., Swanson M.S. A muscleblind knockout model for myotonic dystrophy. Science. 2003; 302:1978–1980. 14671308 10.1126/science.1088583

[62] Chang L. , Zhou G., Soufan O., Xia J. miRNet 2.0: network-based visual analytics for miRNA functional analysis and systems biology. Nucleic Acids Res. 2020; 48:W244–W251. 32484539 10.1093/nar/gkaa467 PMC7319552

[63] Osborne R.J. , Lin X., Welle S., Sobczak K., O’Rourke J.R., Swanson M.S., Thornton C.A. Transcriptional and post-transcriptional impact of toxic RNA in myotonic dystrophy. Hum. Mol. Genet. 2009; 18:1471–1481. 19223393 10.1093/hmg/ddp058 PMC2664149

[64] Mankodi A. , Logigian E., Callahan L., McClain C., White R., Henderson D., Krym M., Thornton C.A. Myotonic dystrophy in transgenic mice expressing an expanded CUG repeat. Science. 2000; 289:1769–1772. 10976074 10.1126/science.289.5485.1769

[65] Du H. , Cline M.S., Osborne R.J., Tuttle D.L., Clark T.A., Donohue J.P., Hall M.P., Shiue L., Swanson M.S., Thornton C.A. et al. Aberrant alternative splicing and extracellular matrix gene expression in mouse models of myotonic dystrophy. Nat. Struct. Mol. Biol. 2010; 17:187–193. 20098426 10.1038/nsmb.1720 PMC2852634

[66] Nunes A.M. , Ramirez M., Jones T.I., Jones P.L. Identification of candidate miRNA biomarkers for facioscapulohumeral muscular dystrophy using DUX4-based mouse models. Dis Model Mech. 2021; 14:dmm049016. 34338285 10.1242/dmm.049016 PMC8405850

[67] Mitra R. , Adams C.M., Jiang W., Greenawalt E., Eischen C.M. Pan-cancer analysis reveals cooperativity of both strands of microRNA that regulate tumorigenesis and patient survival. Nat. Commun. 2020; 11:968. 32080184 10.1038/s41467-020-14713-2 PMC7033124

[68] Li M. , Zhuang Y., Batra R., Thomas J.D., Li M., Nutter C.A., Scotti M.M., Carter H.A., Wang Z.J., Huang X.-S. et al. HNRNPA1-induced spliceopathy in a transgenic mouse model of myotonic dystrophy. Proc. Natl. Acad. Sci. U.S.A. 2020; 117:5472–5477. 32086392 10.1073/pnas.1907297117 PMC7071875

[69] Bailey L.R.J. , Bugg D., Reichardt I.M., Ortaç C.D., Nagle A., Gunaje J., Martinson A., Johnson R., MacCoss M.J., Sakamoto T. et al. MBNL1 regulates programmed postnatal switching between regenerative and differentiated cardiac states. Circulation. 2024; 149:1812–1829. 38426339 10.1161/CIRCULATIONAHA.123.066860 PMC11147738

[70] Yang A. , Bofill-De Ros X., Shao T.-J., Jiang M., Li K., Villanueva P., Dai L., Gu S. 3′ Uridylation confers miRNAs with non-canonical target repertoires. Mol. Cell. 2019; 75:511–522. 31178353 10.1016/j.molcel.2019.05.014 PMC6688926

[71] Fernandez-Valverde S.L. , Taft R.J., Mattick J.S. Dynamic isomiR regulation in *Drosophila* development. RNA. 2010; 16:1881–1888. 20805289 10.1261/rna.2379610 PMC2941097

[72] Muiwo P. , Pandey P., Ahmad H.M., Ramachandran S.S., Bhattacharya A. IsomiR processing during differentiation of myelogenous leukemic cell line K562 by phorbol ester PMA. Gene. 2018; 641:172–179. 29051025 10.1016/j.gene.2017.10.025

[73] Goers E.S. , Purcell J., Voelker R.B., Gates D.P., Berglund J.A. MBNL1 binds GC motifs embedded in pyrimidines to regulate alternative splicing. Nucleic. Acids. Res. 2010; 38:2467–2484. 20071745 10.1093/nar/gkp1209 PMC2853123

[74] Ellis J. , Hale M., Cleary J., Wang E., Berglund J.A. Alternative Splicing Outcomes Across an RNA-Binding Protein Concentration Gradient. J. Mol. Biol. 2023; 435:168156. 37230319 10.1016/j.jmb.2023.168156

[75] Nakamori M. , Sobczak K., Puwanant A., Welle S., Eichinger K., Pandya S., Dekdebrun J., Heatwole C.R., McDermott M.P., Chen T. et al. Splicing biomarkers of disease severity in myotonic dystrophy. Ann. Neurol. 2013; 74:862–872. 23929620 10.1002/ana.23992 PMC4099006

[76] Huang H.-Y. , Lin Y.-C.-D., Li J., Huang K.-Y., Shrestha S., Hong H.-C., Tang Y., Chen Y.-G., Jin C.-N., Yu Y. et al. miRTarBase 2020: updates to the experimentally validated microRNA–target interaction database. Nucleic Acids Res. 2019; 48:D148–D154. 10.1093/nar/gkz896 PMC7145596 31647101

[77] Koehorst E. , Ballester-Lopez A., Arechavala-Gomeza V., Martínez-Piñeiro A., Nogales-Gadea G. The biomarker potential of miRNAs in myotonic dystrophy type I. J. Clin. Med. 2020; 9:3939. 33291833 10.3390/jcm9123939 PMC7762003

[78] Liu B. , Shyr Y., Cai J., Liu Q. Interplay between miRNAs and host genes and their role in cancer. Brief. Funct. Genomics. 2019; 18:255–266. 10.1093/bfgp/elz002 PMC6609535 30785618

[79] Batra R. , Charizanis K., Manchanda M., Mohan A., Li M., Finn D.J., Goodwin M., Zhang C., Sobczak K., Thornton C.A. et al. Loss of MBNL leads to disruption of developmentally regulated alternative polyadenylation in RNA-mediated disease. Mol. Cell. 2014; 56:311–322. 25263597 10.1016/j.molcel.2014.08.027 PMC4224598

[80] Tan G.C. , Chan E., Molnar A., Sarkar R., Alexieva D., Isa I.M., Robinson S., Zhang S., Ellis P., Langford C.F. et al. 5′ isomiR variation is of functional and evolutionary importance. Nucleic. Acids. Res. 2014; 42:9424–9435. 25056318 10.1093/nar/gku656 PMC4132760

[81] Morgan M. , Kabayama Y., Much C., Ivanova I., Di Giacomo M., Auchynnikava T., Monahan J.M., Vitsios D.M., Vasiliauskaitė L., Comazzetto S. et al. A programmed wave of uridylation-primed mRNA degradation is essential for meiotic progression and mammalian spermatogenesis. Cell Res. 2019; 29:221–232. 30617251 10.1038/s41422-018-0128-1 PMC6420129

[82] Gutiérrez-Vázquez C. , Enright A.J., Rodríguez-Galán A., Pérez-García A., Collier P., Jones M.R., Benes V., Mizgerd J.P., Mittelbrunn M., Ramiro A.R. et al. 3′ Uridylation controls mature microRNA turnover during CD4 T-cell activation. RNA. 2017; 23:882–891. 28351886 10.1261/rna.060095.116 PMC5435861

[83] Horak M. , Novak J., Bienertova-Vasku J. Muscle-specific microRNAs in skeletal muscle development. Dev. Biol. 2016; 410:1–13. 26708096 10.1016/j.ydbio.2015.12.013

[84] JU H. , Yang Y., Sheng A., Jiang X. Role of microRNAs in skeletal muscle development and rhabdomyosarcoma (review). Mol. Med. Rep. 2015; 11:4019–4024. 25633282 10.3892/mmr.2015.3275

[85] Mytidou C. , Koutsoulidou A., Zachariou M., Prokopi M., Kapnisis K., Spyrou G.M., Anayiotos A., Phylactou L.A. Age-related exosomal and endogenous expression patterns of miR-1, miR-133a, miR-133b, and miR-206 in skeletal muscles. Front. Physiol. 2021; 12:708278. 34867435 10.3389/fphys.2021.708278 PMC8637414

[86] He C. , Yang J., Ding J., Li S., Wu H., Xiong Y., Zhou F., Jiang Y., Teng L., Yang J. Downregulation of glucose6phosphate dehydrogenase by microRNA1 inhibits the growth of pituitary tumor cells. Oncol. Rep. 2018; 40:3533–3542. 30272333 10.3892/or.2018.6755

[87] Chinchilla A. , Lozano E., Daimi H., Esteban F.J., Crist C., Aranega A.E., Franco D. MicroRNA profiling during mouse ventricular maturation: a role for miR-27 modulating Mef2c expression. Cardiovasc. Res. 2011; 89:98–108. 20736237 10.1093/cvr/cvq264

[88] Lozano-Velasco E. , Contreras A., Crist C., Hernández-Torres F., Franco D., Aránega A.E. Pitx2c modulates Pax3+/Pax7+ cell populations and regulates Pax3 expression by repressing miR27 expression during myogenesis. Dev. Biol. 2011; 357:165–178. 21749861 10.1016/j.ydbio.2011.06.039

[89] Cerro-Herreros E. , González-Martínez I., Moreno-Cervera N., Overby S., Pérez-Alonso M., Llamusí B., Artero R. Therapeutic potential of AntagomiR-23b for treating myotonic dystrophy. Mol. Ther. Nucleic Acids. 2020; 21:837–849. 32805487 10.1016/j.omtn.2020.07.021 PMC7452101

[90] Cerro-Herreros E. , Sabater-Arcis M., Fernandez-Costa J.M., Moreno N., Perez-Alonso M., Llamusi B., Artero R. miR-23b and miR-218 silencing increase muscleblind-like expression and alleviate myotonic dystrophy phenotypes in mammalian models. Nat. Commun. 2018; 9:2482. 29946070 10.1038/s41467-018-04892-4 PMC6018771

[91] Overby S.J. , Cerro-Herreros E., González-Martínez I., Varela M.A., Seoane-Miraz D., Jad Y., Raz R., Møller T., Pérez-Alonso M., Wood M.J. et al. Proof of concept of peptide-linked blockmiR-induced MBNL functional rescue in myotonic dystrophy type 1 mouse model. Mol. Ther. Nucleic Acids. 2022; 27:1146–1155. 35282418 10.1016/j.omtn.2022.02.003 PMC8888893

[92] Shang R. , Lai E.C. Parameters of clustered suboptimal miRNA biogenesis. Proc. Natl. Acad. Sci. U.S.A. 2023; 120:e2306727120. 37788316 10.1073/pnas.2306727120 PMC10576077

[93] Melamed Z. , Levy A., Ashwal-Fluss R., Lev-Maor G., Mekahel K., Atias N., Gilad S., Sharan R., Levy C., Kadener S. et al. Alternative splicing regulates biogenesis of miRNAs located across exon-intron junctions. Mol. Cell. 2013; 50:869–881. 23747012 10.1016/j.molcel.2013.05.007

[94] Agranat-Tamir L. , Shomron N., Sperling J., Sperling R. Interplay between pre-mRNA splicing and microRNA biogenesis within the supraspliceosome. Nucleic Acids Res. 2014; 42:4640–4651. 24464992 10.1093/nar/gkt1413 PMC3985634

[95] Ramalingam P. , Palanichamy J.K., Singh A., Das P., Bhagat M., Kassab M.A., Sinha S., Chattopadhyay P. Biogenesis of intronic miRNAs located in clusters by independent transcription and alternative splicing. RNA. 2014; 20:76–87. 24226766 10.1261/rna.041814.113 PMC3866646

[96] Orbán T.I. One locus, several functional RNAs—Emerging roles of the mechanisms responsible for the sequence variability of microRNAs. Biol. Futur. 2023; 74:17–28. 36847925 10.1007/s42977-023-00154-7

[97] Cappella M. , Perfetti A., Cardinali B., Garcia-Manteiga J.M., Carrara M., Provenzano C., Fuschi P., Cardani R., Renna L.V., Meola G. et al. High-throughput analysis of the RNA-induced silencing complex in myotonic dystrophy type 1 patients identifies the dysregulation of miR-29c and its target ASB2. Cell Death. Dis. 2018; 9:729. 29955039 10.1038/s41419-018-0769-5 PMC6023919

[98] Curcio A. , Torella D., Iaconetti C., Pasceri E., Sabatino J., Sorrentino S., Giampà S., Micieli M., Polimeni A., Henning B.J. et al. MicroRNA-1 downregulation increases connexin 43 displacement and induces ventricular tachyarrhythmias in rodent hypertrophic hearts. PLoS One. 2013; 8:e70158. 23922949 10.1371/journal.pone.0070158 PMC3724819

[99] Magee R. , Telonis A.G., Cherlin T., Rigoutsos I., Londin E. Assessment of isomiR discrimination using commercial qPCR methods. Noncoding RNA. 2017; 3:18. 28730153 10.3390/ncrna3020018 PMC5514785

[100] Nejad C. , Pépin G., Behlke M.A., Gantier M.P. Modified polyadenylation-based RT-qPCR increases selectivity of amplification of 3′-MicroRNA isoforms. Front. Genet. 2018; 9:11. 29416548 10.3389/fgene.2018.00011 PMC5787544

[101] Ayyadevara S. , Thaden J.J., Shmookler Reis R.J. Discrimination of primer 3′-nucleotide mismatch by Taq DNA polymerase during polymerase chain reaction. Anal. Biochem. 2000; 284:11–18. 10933850 10.1006/abio.2000.4635

[102] Peng C.-Y. , Liao Y.-W., Lu M.-Y., Yu C.-H., Yu C.-C., Chou M.-Y. Downregulation of miR-1 enhances tumorigenicity and invasiveness in oral squamous cell carcinomas. J. Formos. Med. Assoc. 2017; 116:782–789. 28089494 10.1016/j.jfma.2016.12.003

[103] Fritegotto C. , Ferrati C., Pegoraro V., Angelini C. Micro-RNA expression in muscle and fiber morphometry in myotonic dystrophy type 1. Neurolog. Sci. 2017; 38:619–625. 10.1007/s10072-017-2811-2 28078570

[104] Souidi A. , Nakamori M., Zmojdzian M., Jagla T., Renaud Y., Jagla K. Deregulations of miR-1 and its target Multiplexin promote dilated cardiomyopathy associated with myotonic dystrophy type 1. EMBO Rep. 2023; 24:e56616. 36852954 10.15252/embr.202256616 PMC10074075

[105] Chal J. , Pourquié O. Making muscle: skeletal myogenesis *in vivo* and *in vitro*. Development. 2017; 144:2104–2122. 28634270 10.1242/dev.151035

[106] Liu N. , Williams A.H., Kim Y., McAnally J., Bezprozvannaya S., Sutherland L.B., Richardson J.A., Bassel-Duby R., Olson E.N. An intragenic MEF2-dependent enhancer directs muscle-specific expression of microRNAs 1 and 133. Proc. Natl. Acad. Sci. 2007; 104:20844–20849. 18093911 10.1073/pnas.0710558105 PMC2409229

[107] Rota R. , Ciarapica R., Giordano A., Miele L., Locatelli F. MicroRNAs in rhabdomyosarcoma: pathogenetic implications and translational potentiality. Mol. Cancer. 2011; 10:120. 21943149 10.1186/1476-4598-10-120 PMC3212852

[108] Kalsotra A. , Wang K., Li P.-F., Cooper T.A. MicroRNAs coordinate an alternative splicing network during mouse postnatal heart development. Genes Dev. 2010; 24:653–658. 20299448 10.1101/gad.1894310 PMC2849122

