# Supplementary material for: MBNL splicing factors regulate the microtranscriptome of skeletal muscles
Source: Nucleic Acids Res. 2024 Sep 11;52(19):12055–73. doi: 10.1093/nar/gkae774 (PMC11514471; doi:10.1093/nar/gkae774)
Supplement: gkae774_Supplemental_Files [file gkae774_supplemental_files.zip › Supplements.pdf]

MBNL splicing factors regulate the microtranscriptome of skeletal muscles

Agnieszka Piasecka 1, Michał W. Szcześniak 2, Michał Sekrecki 1, Arkadiusz Kajdasz 1,3, Łukasz J. Sznajder 1,4, Anna Baud 1, Krzysztof Sobczak 1,*

1 Laboratory of Gene Therapy, Department of Gene Expression, Institute of Molecular Biology and Biotechnology, Faculty of Biology, Adam Mickiewicz University, Uniwersytetu Poznanskiego 6, 61-614 Poznań, Poland

2 Institute of Human Biology and Evolution, Faculty of Biology, Adam Mickiewicz University, Uniwersytetu Poznanskiego 6, 61-614 Poznań, Poland

3 Laboratory of Bioinformatics, Institute of Bioorganic Chemistry, Polish Academy of Sciences, Noskowskiego 12/14, 61-704 Poznan, Poland

4 Department of Chemistry and Biochemistry, University of Nevada, Las Vegas, NV 89154, USA

* To whom correspondence should be addressed. Tel: (+48) 61 829 5958; E-mail: ksobczak@amu.edu.pl

SUPPLEMENTARY FIGURES

Supplementary Figure S1. Differentially expressed miRNA gene network and miRNA GO network analysis.

Supplementary Figure S2. Specificity of miRNA changes to MBNL1 deficiency.

Supplementary Figure S3 Identification of pri-miR-23b, miR-27b, and miR-24-1 in human cells.

Supplementary Figure S4. Representative electropherograms from the sequencing of different pri-miR-23b/-27b/-24-1 isoforms.

Supplementary Figure S5. Identification of pri-miR-23b, miR-27b, and miR-24-1 in mouse cells.

Supplementary Figure S6. The efficiency of the knock-down process in HeLa cells.

Supplementary Figure S7. Functional interaction between MBNL1 and its cis-acting regulatory elements in the pri-miR-23b fragment.

SUPPLEMENTARY TABLES

Supplementary Table S1. miRNAs expressed differentially between WT and *Mbnl1*KO mice

Supplementary Table S2. miRNAs expressed differentially between WT and *Mbnl2*KO mice

Supplementary Table S3. miRNAs deregulated both in *Mbnl1*KO and *Mbnl2*KO mice

Supplementary Table S4. The deregulated genes in Mbnl1KO mice that are the targets of altered miRNAs in the same mouse model.

Supplementary Table S5. miRNAs expressed differentially between WT and *HSA*-LR mice

Supplementary Table S6. miRNAs deregulated in *Mbnl1*KO mice at the transcript level

Supplementary Table S7. Developmentally regulated miRNA missregulated in *Mbnl1*KO

Supplementary Table S8. miRNAs expressed differentially between non-DM1 and DM1 patients

Supplementary Table S9. List of primers used in this study

Supplementary Table S10. List of siRNAs used in this study

Supplementary Tables S1,S2,S4,S5,S6,S7,S8 are attached as a single Excel file.

Supplementary Tables S3,S9,S10 are included in this document.

SUPPLEMENTARY FIGURES

Fig.S1

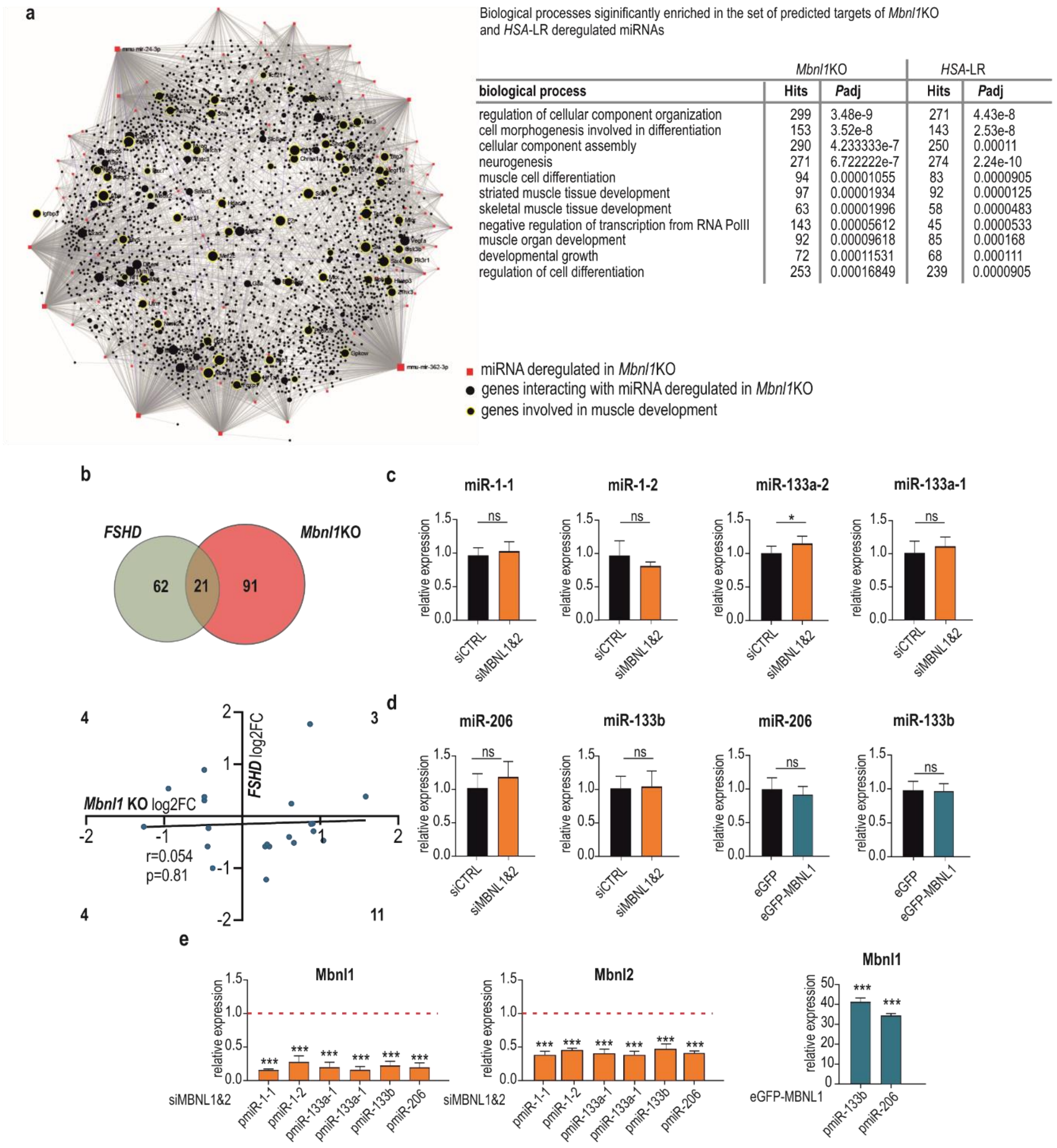

	*Mbnl1*KO		*HSA*-LR	
**biological process**	**Hits**	***Padj***	**Hits**	***Padj***
regulation of cellular component organization	299	3.48e-9	271	4.43e-8
cell morphogenesis involved in differentiation	153	3.52e-8	143	2.53e-8
cellular component assembly	290	4.233333e-7	250	0.00011
neurogenesis	271	6.722222e-7	274	2.24e-10
muscle cell differentiation	94	0.00001055	83	0.0000905
striated muscle tissue development	97	0.00001934	92	0.0000125
skeletal muscle tissue development	63	0.00001996	58	0.0000483
negative regulation of transcription from RNA PolII	143	0.00005612	45	0.0000533
muscle organ development	92	0.00009618	85	0.000168
developmental growth	72	0.00011531	68	0.000111
regulation of cell differentiation	253	0.00016849	239	0.0000905

Figure S1. Differentially expressed miRNA gene network and miRNA GO network analysis (Related to the main Fig. 1).

a) Visualization of the network of miRNAs deregulated in *Mbnl1*KO mice and their target gene interactions; the table presents the results of GO enrichment analysis according to biological process

for targets of deregulated miRNAs in *Mbnl1*KO and *HSA*-LR mice. The analysis was performed with the web-based platform miRNet (https://www.mirnet.ca/home.xhtml).

b) The Venn diagram depicts the proportion of differentially expressed miRNAs in mouse with loss of MBNL1 or FSHD mouse model; there is only 18% overlap in miRNA changes between Mbnl1KO and FSHD mice; the graph below shows that in the group of 22 miRNAs altered in the Mbnl1KO and FSHD model, only 7 are altered in the same direction. Pearson's r= 0.054; p= 0.81 (not significant)

c) MyomiR-1 levels generated from constructs in HeLa cells upon siMBNL1&2 knockdown as determined by real-time RT-PCR and normalized to the levels of U6. Statistical significance was calculated in reference to the control (siCTRL) using Student's t test; ns, nonsignificant, * P < 0.05; the level of clustered miR-133a is presented as well.

d) MyomiR-206 levels generated from constructs in HeLa cells upon siMBNL1&2 knockdown and upon MBNL1 overexpression, as determined by real-time RT-PCR and normalized to the levels of U6. Statistical significance was calculated in reference to the control (siCTRL) using Student's t test; ns, nonsignificant; the level of clustered miR-133b is presented as well.

e) The graphs show knockdown efficiency and overexpression levels of MBNL1 and MBNL2 in HeLa cells with the introduction of myomiR expression plasmids. The levels of MBNL1 and MBNL2 were compared to those observed in HeLa cells treated with a control siRNA or an eGFP plasmid. (* P < 0.05, ** P < 0.01, *** P < 0.001, unpaired t-test).

Fig. S2

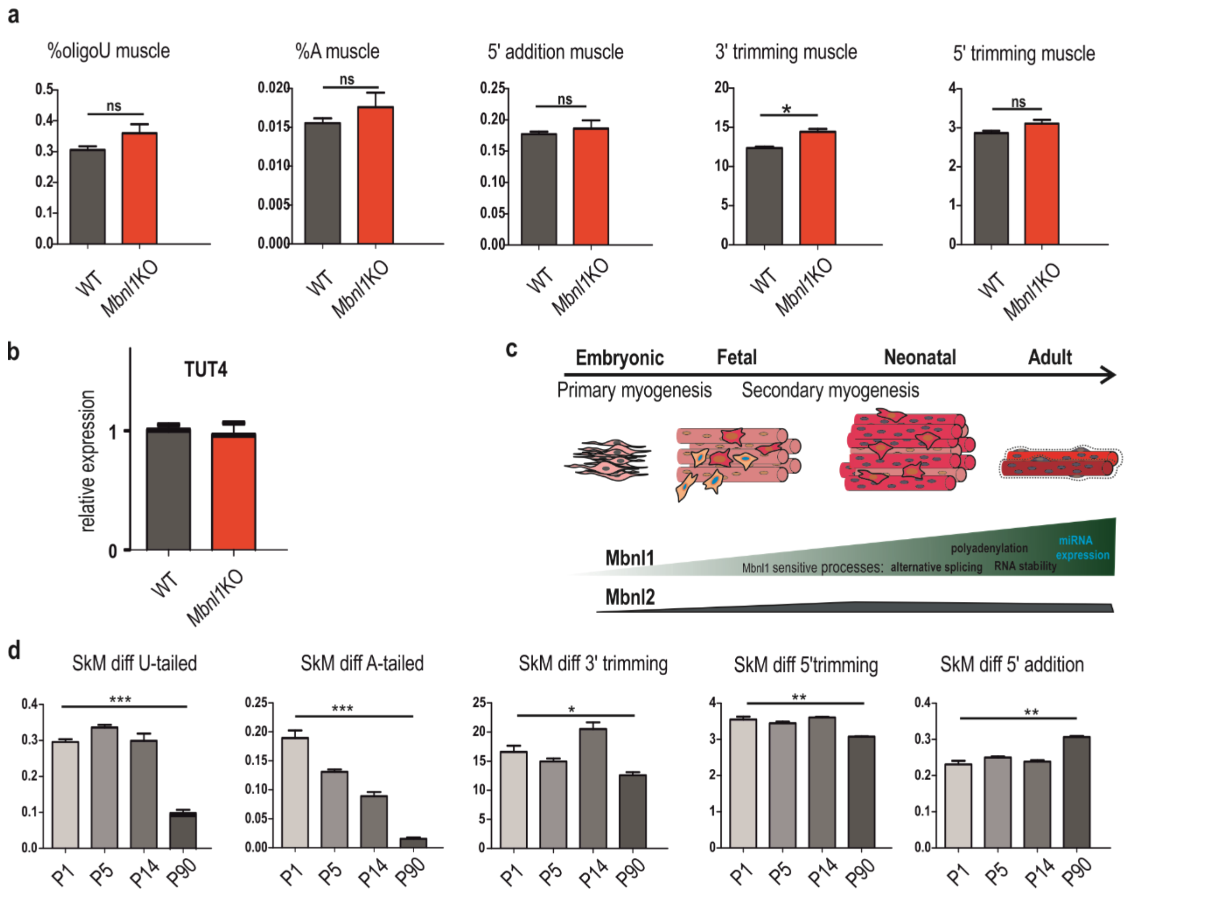

Figure S2. Specificity of miRNA changes to MBNL1 deficiency.

a) Comparison of the frequency of 5'- and 3'-modifications of miRNAs in muscles of WT and Mbnl1KO mice based on the results of small RNA-seq experiments. Statistical significance was calculated in reference to WT mice using Student's t test; ns, nonsignificant, * for $P < 0.05$.

b) Results of real-time PCR analysis of Tut4 expression in Mbnl1KO mice; the result is the average from three independent experiments +/- SD normalized to the expression of Gapdh.

c) Schematic illustration of skeletal muscle formation and MBNL protein levels and activity changes during this process. Some elements of the figure were adapted from Chal J, Pourquie O, 2017

d) Comparison of the frequency of 5'- and 3'-modifications of miRNAs in mouse muscles during postnatal development based on the results of small RNA-seq experiments; SkM diff, skeletal muscle differentiation. Statistical significance was calculated in reference to P1 using two-way Anova; ns, nonsignificant, *for $P < 0.05$, ** for $P < 0.01$, *** for $P < 0.001$.

Fig. S3

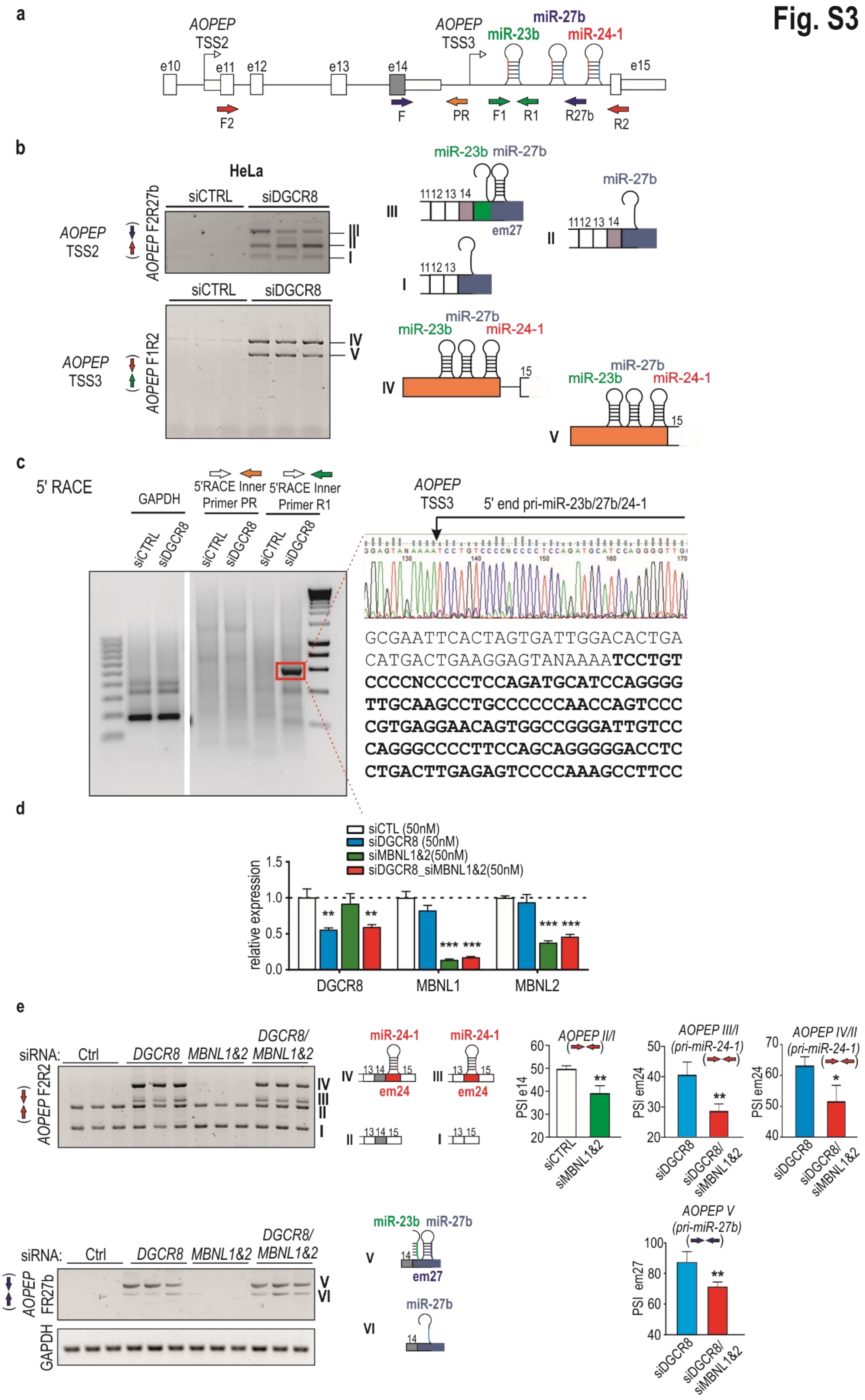
a
AOPEP TSS2
AOPEP TSS3
miR-27b
miR-23b
miR-24-1
e10 e11 e12 e13 e14 e15
F2 F PR F1 R1 R27b R2
b
HeLa
siCTRL siDGCR8
AOPEP TSS2
AOPEP F2R27b
III II I
AOPEP TSS3
AOPEP F1R2
IV V
em27
c
5' RACE
GAPDH
5'RACE Inner Primer PR
5'RACE Inner Primer R1
siCTRL siDGCR8
AOPEP TSS3
5' end pri-miR-23b/27b/24-1
GCGAATTCACTAGTGATTGGACACTGA
CATGACTGAAGGAGTANAAAATCCTGT
CCCCNCCCCTCCAGATGCATCCAGGGG
TTGCAAGCCTGCCCCCCAACCAGTCCC
CGTGAGGAACAGTGGCCGGGATTGTCC
CAGGGCCCCTTCCAGCAGGGGGACCTC
CTGACTTGAGAGTCCCCAAAGCCTTCC
d
siCTL (50nM)
siDGCR8 (50nM)
siMBNL1&2(50nM)
siDGCR8_siMBNL1&2(50nM)
relative expression
DGCR8 MBNL1 MBNL2
e
siRNA: Ctrl DGCR8 MBNL1&2 DGCR8/MBNL1&2
AOPEP F2R2
AOPEP FR27b
GAPDH
em24
AOPEP II/I
PSI e14
siCTRL siMBNL1&2
AOPEP III/I (pri-miR-24-1)
AOPEP IV/II (pri-miR-24-1)
PSI em24
siDGCR8 siDGCR8/siMBNL1&2
AOPEP V (pri-miR-27b)
PSI em27

Figure S3. Identification of pri-miR-23b, miR-27b, and miR-24-1 in human cells (Related to Figure 4).

a) Scheme of a fragment of AOPEP pre-mRNA with marked positions of miR-23b, miR-27b, and miR-24-1 and primers used in the analysis marked with arrows; alternatively spliced ex14 is marked with a gray box.

b) The gels show transcripts identified as a substrate for Drosha in human HeLa cells. Band I on the upper gel represents a transcript consisting of exons of AOPEP and a retained sequence of an intron with part of miR-27b, band II has the same sequence and additionally alternative exon 14 of AOPEP, and band III consists of exons of AOPEP and a retained sequence of an intron with miR-27b and part of miR-23b. Another Drosha-sensitive RNA (lower gel) included nearly the whole intron with miR-23b, miR-27b, and miR-24 sequences (pri-miR-23b/27b/24-1).

c) 5' RLM-RACE PCR and Sanger sequencing results of pri-miR-23b/27b/24-1. GAPDH was amplified as a positive control. As expected, the level of the transcript did not change after siDGCR8-mediated knockdown; pri-miR-23b/27b/24-1 was amplified with two different reverse primers: the first located 700 bp upstream of miR-23b (PR primer) and the second located downstream of miR-23b (R1 primer). The clear product was detected only when the reaction was performed with the R1 primer. Sanger sequencing results revealed that pri-miR-23b/27b/24-1 starts 529 bp upstream of miR-23b.

d) DGCR8, MBNL1 and MBNL2 mRNA levels in HeLa cells upon siDGCR8 or siMBNL1&2 or siDGCR8 and siMBNL1&2 treatment, as determined by RT-qPCR expression analyses; all data for mRNA were averages from three independent experiments +/- SDs normalized to GAPDH mRNA expression (* $P < 0.05$,** $P < 0.01$,*** $P < 0.001$; , unpaired t-test).

e) The level of human mRNA of AOPEP (coordinates: hg38 chr9:94,726,712-95,087,159) was not Drosha sensitive when exons with miRNA sequences were absent (RT–PCR bands I and II). In cells with Drosha knockdown, two additional PCR bands appeared (bands III and IV; upper panel), representing transcripts with alternative exons that included the miR-24-1 sequence. The inclusion of exon 14 of AOPEP and exon em24 (alternative exon with miR-24-1 sequence) was positively regulated by MBNL. Alternatively, spliced pri-miRNA isoforms, which included miR-27b, were also MBNL sensitive (PCR band V; lower panel). The colors of the bars on the graphs correspond to the legend depicted in Fig. S3d. The PSI values for em24 and em27 are depicted in the graph (** $P < 0.01$, *** $P < 0.001$, unpaired t-test).

Fig.S4

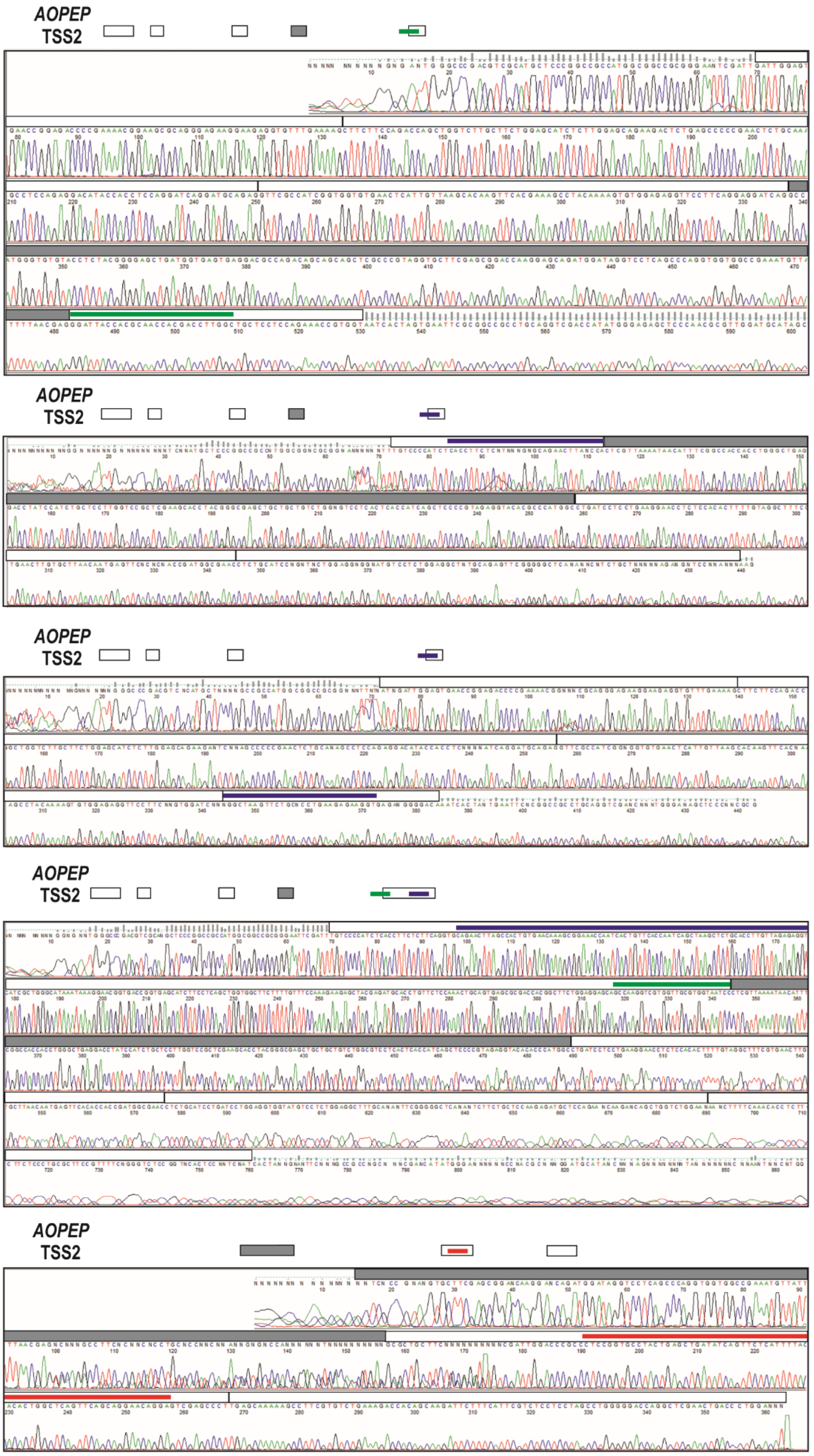

Figure S4. Representative electropherograms from the sequencing of different pri-miR-23b/-27b/-24-1 isoforms (Related to Figure 4).

Above each electropherogram, the pri-miRNA exon composition is depicted; constitutive exons of AOPEP are marked with white boxes, alternatively spliced ex14 is marked with a gray box, pre-miR-23b is marked with a green box, pre-miR-27b is marked with a blue box, and pre-miR-24-1 is marked with red box.

Fig. S5

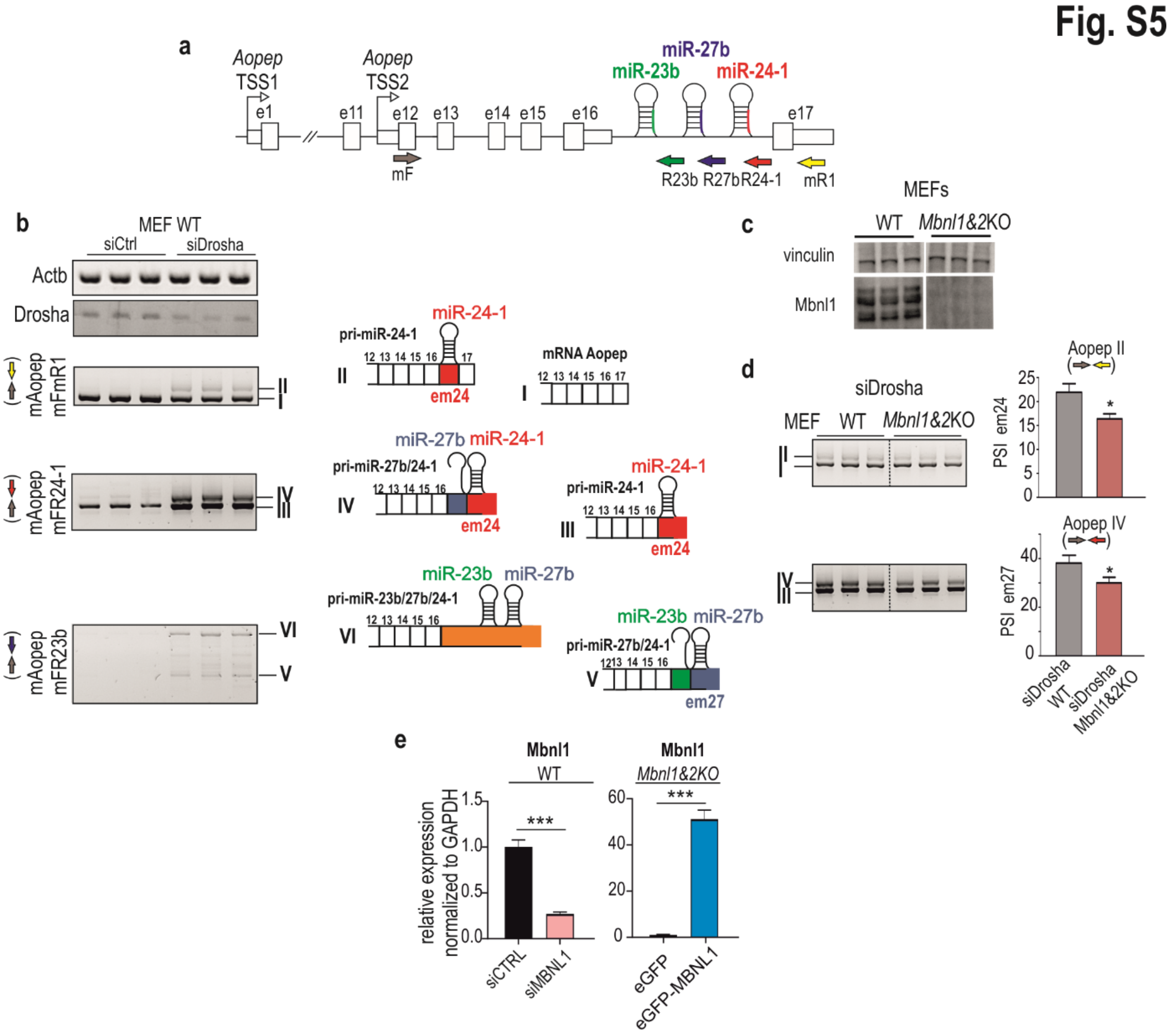

Figure S5. Identification of pri-miR-23b, miR-27b, and miR-24-1 in mouse cells (Related to Fig. 4).

a) To examine whether the pattern of miR-23b, miR-27b, and miR-24 expression is conserved among mammals, we studied pri-miR-23b/27b/24 structure in MEFs. A schematic of a fragment of the Aopep pre-mRNA is shown with the positions of miR-23b, miR-27b, miR-24-1 marked and the primers used in the analysis marked with arrows.

b) Amplification of the mRNA Aopep in siCTRL MEF_WT and in siDrosha MEF_WT (mAopep mFmR1) suggested that the mature transcript composed of constitutional exons (depicted in the figure with white rectangles) was not a substrate for Drosha (band I). However, after Drosha knockdown, an additional band appeared (band II), which represented mRNA with one new exon with a pre-miR-24-1

sequence. Using various reverse primers, we revealed another three pri-miRNAs: (i) mAopep mFR24-1 band IV, which included two new exons, namely, one with part of pre-miR-27b and the second with the miR-24-1 precursor sequence; (ii) mAopep mFR23b band VI, which included the miR-23b and miR-27b sequences and the miR-27b sequence but not the full sequence of miR-23b (band V). Three pri-miRNAs consisted of all Aopep exons plus additional exons with miR-27b or miR-24-1 sequences (pri-miR-27b/24-1 and pri-miR-24). The fourth primary pri-miRNA included the whole intron with miR-23b, miR-27b and probably miR-24 sequences (pri-miR-23b/27b/24-1). From primary miR-24-1, only one miRNA could be generated (miR-24-1). From pri-miR-27b/24-1, theoretically, two miRNAs could arise (miR-27b and miR-24-1). From pri-miR-23b/27b/24-1, three miRNAs (miR-23b, miR-27b and miR-24-1) could arise.

c) Western blots demonstrating the abundance of Mbnl1 protein in MEFs of the wild-type (WT) and *Mbnl1&2* knockout (KO) genotypes.

d) Representative gels and calculations of RT-PCR analyses in WT and *Mbnl1&2*KO MEFs (n = 3) on Drosha knockdown background showing that Mbnl1&2 depletion promoted exclusion of exons with miR-24-1 and miR-27b sequences. Statistical significance was calculated in reference to WT MEFs using Student's t test; ns, nonsignificant, for (* $P < 0.05$,** $P < 0.01$,*** $P < 0.001$).

e) Mbnl1 mRNA levels in MEF cells upon siMBNL1 treatment and in MEF_ *Mbnl1&2*KO upon MBNL1 overexpression. mRNA levels were determined by RT-qPCR expression analyses; all data for mRNA were averages from three independent experiments +/- SDs normalized to GAPDH mRNA expression (* $P < 0.05$,** $P < 0.01$,*** $P < 0.001$; , unpaired t-test). Data complements results presented on Fig.4D.

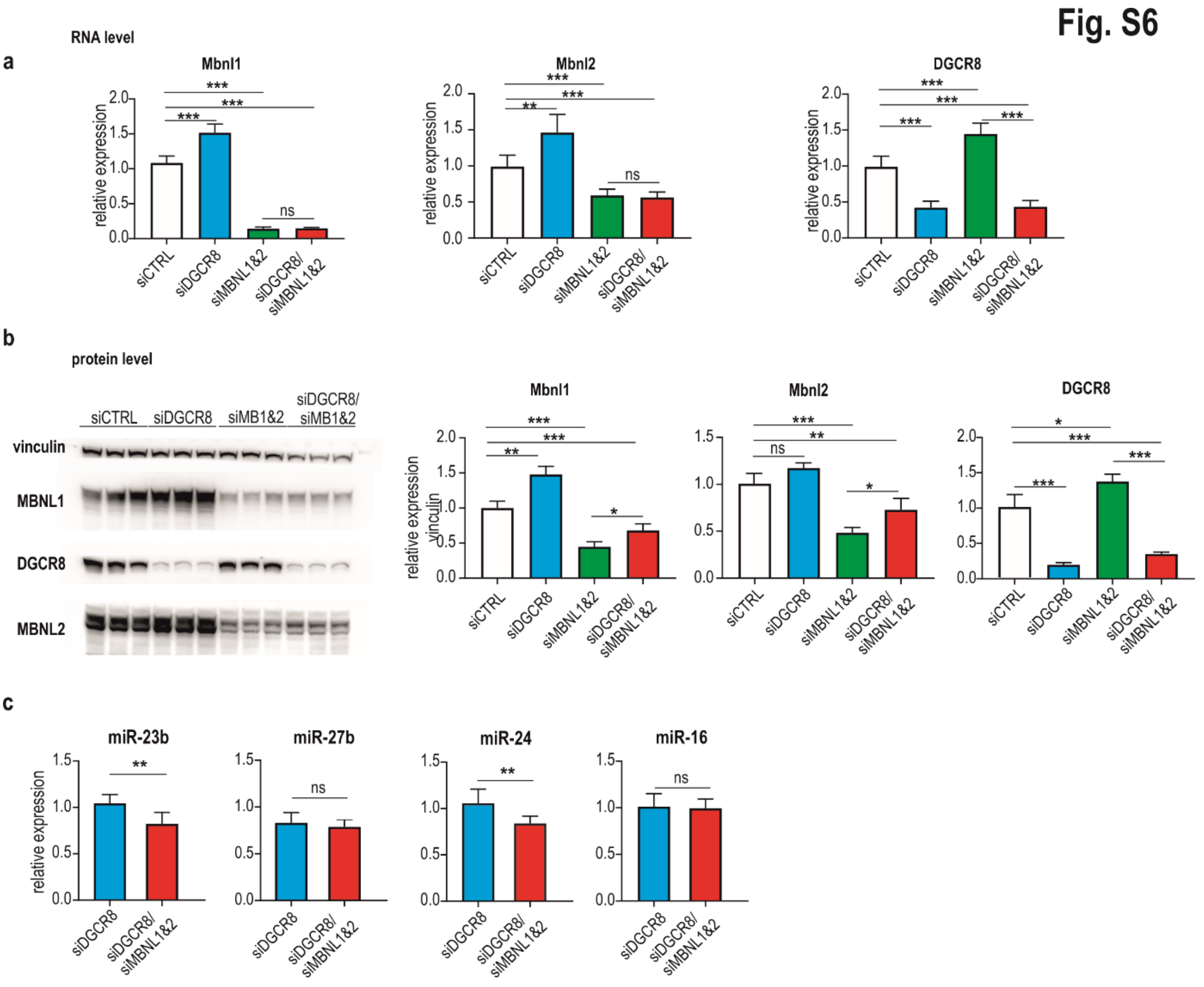

Figure S6. The efficiency of the knock-down process in HeLa cells (Related to Fig. 4).

a) DGCR8, MBNL1 and MBNL2 mRNA levels in HeLa cells upon siDGCR8 or siMBNL1&2 or siDGCR8 and siMBNL1&2 treatment, as determined by RT-qPCR expression analyses; all data for mRNA were averages from three independent experiments +/- SDs normalized to GAPDH mRNA expression (* $P < 0.05$,** $P < 0.01$,*** $P < 0.001$; , unpaired t-test). Data complements results presented on Fig.4B.

b) The same as in S6a but at the protein level. The graphs illustrate the calculation of the results presented on the western blots. Data complements results presented on Fig.4B.

c) miRNAs levels in HeLa cells upon siDGCR8 or siDGCR8 and siMBNL1&2 treatment, as determined by RT-qPCR expression analyses; all data for mRNA were averages from three independent experiments +/- SDs normalized to U6 (* $P < 0.05$,** $P < 0.01$,*** $P < 0.001$; , unpaired t-test). Data complements results presented on Fig.4B.

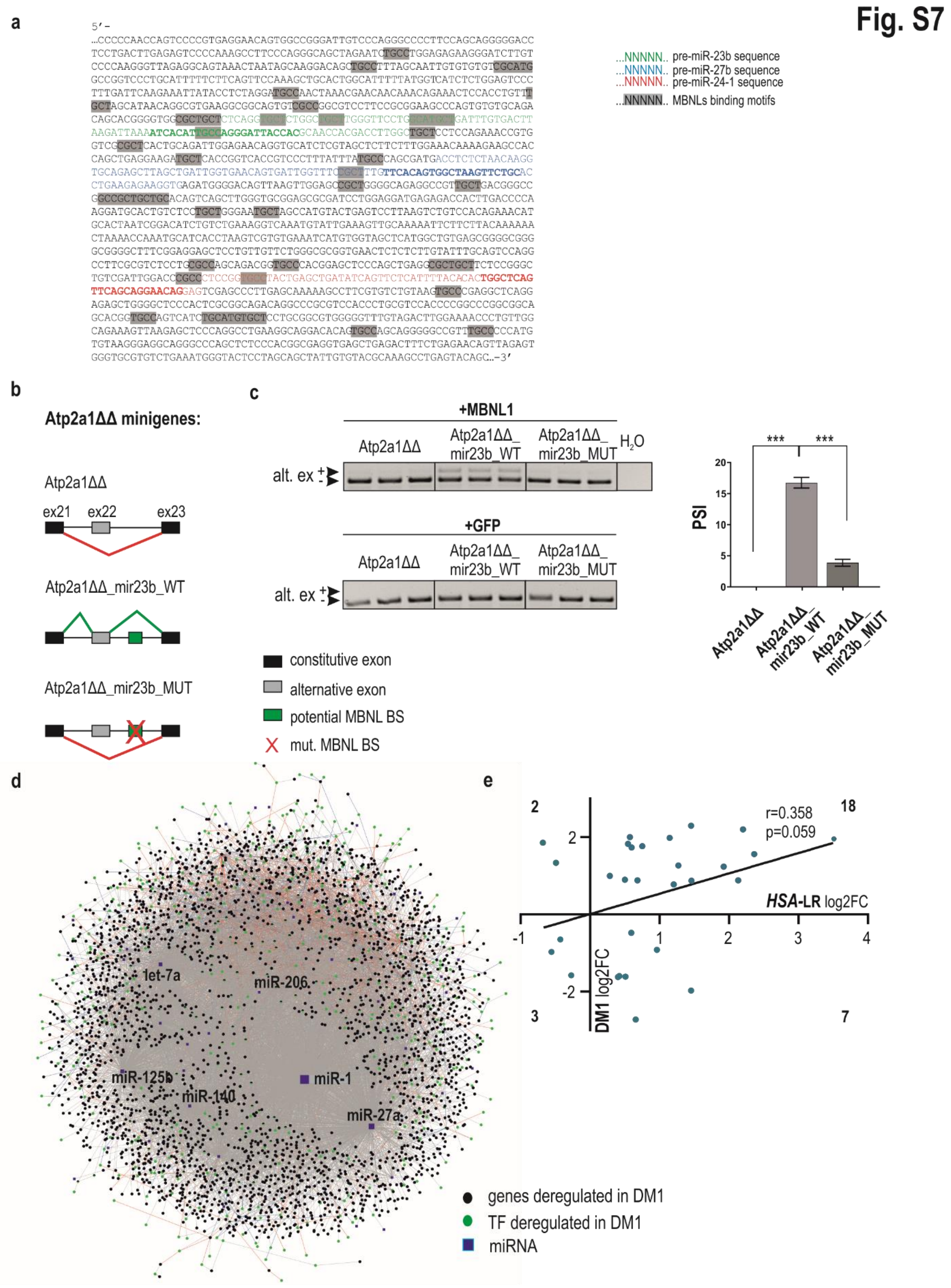

Figure S7. Functional interaction between MBNL1 and its cis-acting regulatory elements in the pri-miR-23b fragment (Related to Fig. 5).

a) Fragment of the AOPEP sequence that includes pre-miR-23b, pre-miR-27b and pre-miR-24-1 with the indicated potential MBNL1 binding sites, which are highlighted in gray. Pre-miR-23b is marked in

green, pre-miR-27b is marked in blue, and pre-miR-24-1 is marked in red. Mature miRNA sequences are bolded.

b) Scheme of three Atp2a1e22 splicing minigenes, Atp2a1ΔΔ, Atp2a1ΔΔ_23b_WT and Atp2a1ΔΔ_23b_MUT without MBNL-binding sites, with the fragment of pri-miR-23b and with the fragment of pri-miR-23b in which potential MBNL-binding sites were mutated, respectively. MBNL BS is the MBNL-binding site; ΔΔ is deletion of the MBNL-binding site.

c) Representative gels with bands representing isoforms with and without exon 22 and quantification of RT-PCR analyses showing a response of Atp2a1e22 minigenes to MBNL1 overexpression. GFP overexpression was a control. The data represent the mean PSI values +/- SDs (n = 3). Statistical significance was calculated in reference to Atp2a1ΔΔ_23b_MUT (*** $P < 0.001$).

d) Visualization of the network of genes deregulated in muscle biopsies of DM1 patients and miRNAs that control them. It was prepared with the web-based platform miRNet (https://www.mirnet.ca/home.xhtml). The list of deregulated genes comes from microarray expression experiments described in Nakamori M et al. Ann Neurol. 2013.

e) The graph presents a comparison of miRNA expression changes in two sets of experiments: *HSA*-LR mice vs. patients with DM1. The scatter plots of miRNA log2FC expression values were created by comparison of the same 30 miRNAs significantly changed in *HSA*-LR mice (Padj < 0.05) and in muscle biopsies of DM1 patients. Pearson’s r= 0.358; p= 0.059. Data complements results presented on Fig. 5A.

SUPPLEMENTARY TABLES

Supplementary Table S3

miRNAs deregulated both in *Mbnl1*KO and *Mbnl2*KO mice

miRNA name	baseMean	log2FoldChange	pvalue	padj
mmu-miR-148a-3p	3818,903556	0,705403912	6,13E-07	0,000146
mmu-miR-101b-3p	4572,221265	-0,583124255	9,14E-07	0,000146
mmu-miR-203-3p	326,629437	0,731914957	2,71E-06	0,000217
mmu-miR-181b-5p	2653,693752	-0,464455485	2,25E-06	0,000217
mmu-miR-24-3p	30298,36836	0,261930956	5,84E-05	0,002336
mmu-miR-379-5p	2062,276998	0,465899234	0,000156	0,005552
mmu-miR-100-5p	8042,617716	-0,46027513	0,000182	0,005835
mmu-miR-182-5p	85,24528774	1,102486168	0,000252	0,006723
mmu-miR-206-3p	7388,118357	0,629836379	0,000446	0,010984
mmu-miR-24-2-5p	2407,06723	0,307543174	0,001908	0,035907
mmu-let-7d-3p	3402,753944	-0,363962464	0,002131	0,037881

Supplementary Table S9

List of primers used in this study

Name of a primer	Sequence (5'>3')
GAPDH_F	GAGTCAACGGATTTGGTCGT
GAPDH_R	TTGATTTTGGAGGGATCTCG
mGapdh_F	ACAGTCCATGCCATCACTGC
mGapdh_R	GCCTGCTTCACCACCTTCTT
mAtp2a1_F	GCTCATGGTCCTCAAGATCTCAC
mAtp2a1_R	GGGTCAGTGCCTCAGCTTTG
mLdb3_F	ACCCTGATGAAGAGGCTCTGCG
mLdb3_R	TGGACCCTCGCTGTAGCTGGTA
mSlain_F	ATACCTCGAATGCAGCCTCAG
mSlain_R	GGTGAGGAGGTGGTTTGTGT
hMBNL1_F	GATCTTGCCGACTGCACCAATG
hMBNL1_R	TTGCCACGTTGGTACTCTCGAC
hMBNL2_F	ATTCTGCCCACCACGCCTGTTA
hMBNL2_R	GGCACAGTTTCCTCGCTGGAAC
mMbnl1_F	CAAGCCTGGTTCCAGCAGAGAT
mMbnl1_R	AATTGCCACGCTGGTACTCTCG
mMbnl2_F	AGTCCCAACAGAGGTTCTACCC
mMbnl2_R	GGCACAGTTTCCTCGCTGGAAC
MEF2C_F	ATATGCAAGCAAAATCTCCTCC
MEF2C_R	GTTGCTACGGAAAACCACTGGGGTA
mMef2c_F	GTCTGATGGGCGGAGATCTG
mMef2c_R	CTTGCTGCCAGGTGGGATAA
pri-miR-1-1_F	GGCAGTAGACTCCAGGGAAG
pri-miR-1-1_R	GACACAGGCAAAGTGACAGAAC

pri-miR-1-1_F2	AGAGCTTGAGGGAAACTCCAC
pri-miR-1-1_R2	ATCATCAGCAACGCTGACTC
pri-miR-133a-2_F	AGAGCTTGAGGGAAACTCCAC
pri-miR-133a-2_R	GACAGTCGATTTGGTTCCATTT
pri-miR-1-2_F	GCAAAAAGAATCAAACCAGGAC
pri-miR-1-2_R	CATTCCATAGCATTGTATGTTCA
pri-miR-1-2_F2	CTCATCCTGGTTTTTCTCCTTG
pri-miR-1-2_R2	AAGCATGCAGAAAGTCATAAGC
pri-miR-133a-1_F	GCAGGAAAACAGTAGGAAAGTG
pri-miR-133a-1_R	ACAAATGAAAACGTTGGTTGTC
pri-miR-206_F	TCCCAGTGATCTTCTCGCTAAG
pri-miR-206_R	GGAGATAGGGGTGTTCAGGAAG
pri-miR-133b_F	ACACACCAAGATACCTGCACAC
pri-miR-133b_R	TGACTCCAGGACTCCTCTTCTC
mAopep_TS1F	ACTTTCCTCGTGCCATCAGA
mAopep_TS1R	CTGTAGCCTGCCATGTTGAC
mAopep_TS2F	AATGGATTCGAGTCAACCGC
mAopep_TS2R	AGATGGTAAGTCTGCTGGAGA
hAopep_TS1F	GTCAACATGGCAGGCTACAG
hAopep_TS1R	AGAAGGGTCTCTCTGTTGCC
hAopep_TS2F	GCTGGTCTTGCTTCTGGAG
hAopep_TS2R	GGCTTTCGTGAACTTGTGCT
23bFh	GCTGCTCTCAGGTGCTCTG
23bRh	ACCACGGTTTCTGGAGGAG
27bFh	CCCAGCGATGACCTCTCTAA
27bRh	TGTCCCCATCTCACCTTCTC
24-1Fh	CGGTGCCTACTGAGCTGAT
24-1Rh	CCTCGGGCACTTACAGACAC
hEx23b_F	TTTTAACGAGGGATTACCACGCA
hEx23b_R	ACGGTGACCGGTGAGCATCTT
hEx27bs_F	AGGAGGATCAGTGGCTAAGTTCT
hEx27bl_F	ATTTTAACGAGTGGCTAAGTTCT
mEx23b_F	TTCTAAAGAGggattaccacgc
mEx27b_F	TTCTAAAGAGtggctaagttct
mEx27b24_F	CTGAAGAGAAGTCCAGGTCTC
mEx24_F	TTCTAAAGAGTCCAGGTCTCC
23bFm	ACATGTGGATGGGAGTGGTT
23bRm	GCCTCTGTTCTCCAAACTGC
27bFm	AGATGCTCACCAGCCCTTTA
27bRm	TGTCCCCATCTCACCTTCTC
24-1Fm	TCTCCATGTCTCCACAGTCG
24-1Rm	ATGGGTAGGCTTTTTGCTCA
hE13e24_F	AGGAGGATCAGCTTCGCGTCTC
hE14e24_F	ATTTTAACGAGCTTCGCGTCTC
hE14e15_F	ATTTTAACGAGGAAAGACCACA
hE13e15_F	AGGAGGATCAGGAAAGACCACA
mE16e17_F	TTCTAAAGAGGACAGATCACA
miR-16	AGCAGCACGTAAATATTGGC
U6	GGATGACACGCAAATTCGTG
18S	GATGGTAGTCGCCGTGCC
5S	CATACCACCCTGAACGCG
miR-1	TGGAATGTAAAGAAGTATGT
miR-206	TGGAATGTAAGGAAGTGTGT

miR-133a	TGGTCCCCTTCAACCAGCTG
miR-133b	TGGTCCCCTTCAACCAGCTA
miR-27b	TTCACAGTGGCTAAGTTCTG
miR-23b	TCACATTGCCAGGGATTAC
miR-24	GGCTCAGTTCAGCAGGAACA
anchored oligo(dT)	GTGCAGGGTCCGAGGTTCAACTATAGGTTTTTTTTTTTTTTT TTTTTTTTTVN
universal Reverse	GTGCAGGGTCCGAGGT
Atp2a1minigenF	GATCTTCAAGCTCCGGGCCCTG
Atp2a1minigenR	AGCAATCAGCTAGTCAGTTGCC

Supplementary Table S10

siRNAs sequences

siRNA	Sequence (5’>3’)
sihMBNL1_	P-rUrCrUrCrUrArCrArUrArCrUrUrCrCrArGrUrGdTdT
sihMBNL2	P-rGrArGrCrGrUrGrArGrCrArUrGrUrUrCrCrUrCdTdT
simMbnl1	P- rCrArCrUrGrGrArArGrUrArUrGrUrArGrArGrAdTdT
simMbnl2_SMARTpool	Dharmacon; a mixture of 4 siRNA provided as a single reagent
siCTRL_s	P-rUrCrGrArArGrUrArUrUrCrCrGrCrGrUrArCrGdTdT
siCTRL_as	P-rCrGrTrArCrGrCrGrGrArArTrArCrTrTrCrGrAdTdT
sihDGCR8_SMARTpool	Dharmacon; a mixture of 4 siRNA provided as a single reagent
simDrosha_SMARTpool	Dharmacon; a mixture of 4 siRNA provided as a single reagent
